# Thermally Activated Delayed Fluorescence Hybrid Copper(I) Iodide Scintillator for Fast Neutron and X‑ray Imaging

**DOI:** 10.1021/jacs.6c03617

**Published:** 2026-04-21

**Authors:** Qingsong Hu, Zizhen Bao, Hadeer Elsayed, Jian-Xin Wang, Linyue Liu, Jiawen Xiao, Guangda Niu, Wentao Wu, Tengyue He, Murilo C Faleiros, Bashir E Hasanov, Yan Jiang, Chengkai Zhang, Di Sun, Osman M. Bakr, Omar F. Mohammed

**Affiliations:** 1 Materials Science & Applied Physics Department, Division of Physical Science and Engineering (PSE), 127355 King Abdullah University of Science and Technology (KAUST), Thuwal 23955-6900, Saudi Arabia; 2 Hubei Key Laboratory of Low Dimensional Optoelectronic Materials and Devices, Hubei University of Arts and Science, Xiangyang 441053,China; 3 School of Physics and Astronomy, 47836 Beijing Normal University, Beijing 100875, P. R. China; 4 School of Nuclear Science and Technology, Xi’an Jiaotong University, Xi’an 710049, China; 5 State Key Laboratory of Intense Pulsed Radiation Simulation and Effect, 506395 Northwest Institute of Nuclear Technology, Xi’an 710024, China; 6 Beijing Key Lab of Microstructure and Property of Solids, College of Materials Science and Engineering, 12496 Beijing University of Technology, Beijing 100124, China; 7 Wuhan National Laboratory for Optoelectronics and School of Optical and Electronic Information, Optical Valley Laboratory, 12443 Huazhong University of Science and Technology, Wuhan 430074, China; 8 Experimental Center for Advanced Materials, School of Materials Science and Engineering, Beijing Institute of Technology, Beijing 100081, China; 9 School of Chemistry and Chemical Engineering, State Key Laboratory of Crystal Materials, 12589 Shandong University, Ji’nan 250100, People’s Republic of China

## Abstract

Developing high-performance dual imaging applications, such as fast neutron and X-ray applications, using a single material presents a very significant challenge across chemistry, material science, physics, and engineering. Integrating both imaging capabilities into a single material for specialized detection applications will simplify device design and significantly reduce overall detection costs. This work represents the first demonstration of a lead-free system designed for high-performance dual imaging applications. It features a multifunctional hybrid copper­(I) iodide scintillator, in which hydrogen-rich and luminescent units are synergistically coupled at the molecular level, enabling the simultaneous imaging of fast neutrons and X-rays. The perfect synergy of exciton confinement and thermally activated delayed fluorescence (TADF) effects empowers this material with exceptional dual imaging capabilities. The confined structure formed by heavy-atom modules at the core imparts a high exciton binding energy, suppressing the nonradiative recombination of excitons. The TADF mechanism channels phonons generated by high-energy radiation into the radiative recombination process. Additionally, the lack of self-absorption guarantees efficient photon utilization. Leveraging these properties, the material achieves an impressive X-ray light yield of approximately 42,000 photons/MeV and an exceptional spatial resolution of 25.8 lp/mm for X-ray imaging, surpassing most commercial scintillators available in the X-ray market. Furthermore, the material demonstrates an outstanding spatial resolution of 1.47 lp/mm in fast neutron imaging, representing the best level reported to date for a Pb-free scintillator. This environmentally friendly and high-performance multifunctional scintillator significantly advances next-generation scintillation materials, presenting exciting opportunities for high-precision and dual imaging applications at a low cost.

## Graphical abstract

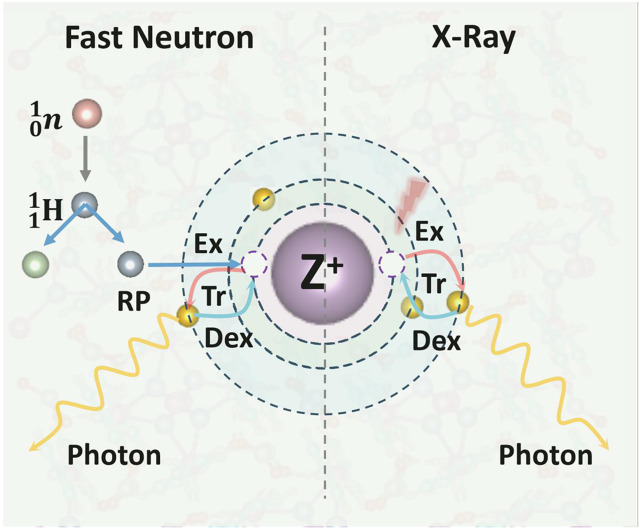

## Introduction

Fast neutron imaging uses high-energy neutrons (typically ≥ 0.1 MeV) to penetrate objects and generate images. This technique is valuable in specialized application scenarios, including detecting light-element materials in explosives and drugs, analyzing defects in composite materials, inspecting internal structures in nuclear fuel elements, and nondestructively examining historical artifacts.^1–5^ Fast neutron imaging offers several advantages over other imaging techniques, including X-ray imaging. These benefits include increased sensitivity to light elements, enhanced penetration, and the capacity to identify internal defects in complex structures.^6,7^ However, this approach also has notable drawbacks, including the high cost of equipment, challenges in neutron source acquisition, relatively low imaging resolution, and stringent safety requirements.^8^ In contrast, X-ray imaging, the most widely used nondestructive testing technology, has extensive applications in medical imaging, industrial inspection, security screening, and material analysis.^9–12^ X-ray imaging has high-resolution, mature, and cost-effective equipment, and the capability of real-time dynamic imaging.^8^ Nevertheless, the limitations of X-ray imaging include insensitivity to light elements (e.g., hydrogen [H]), difficulty penetrating high-density materials, and susceptibility to scattering effects, which can reduce image contrast, sensitivity, and spatial resolution.^13^ Due to their distinct physical properties, fast neutron and X-ray imaging often complement complex inspection tasks. Combining these two imaging modalities enables more comprehensive and accurate detection in various fields, such as aerospace material testing, security screening, and nuclear industry applications.^14–17^ Table S1 compares the advantages and limitations of fast neutron and X-ray imaging.

Scintillators are critical in fast neutron and X-ray imaging because their performance determines image quality and detection efficiency. Significant advancements have recently been achieved in scintillator materials for both imaging techniques.^13,18–21^ However, research on scintillators for fast neutron imaging remains relatively limited. Traditional fast neutron scintillators primarily include organic scintillators (e.g., plastic scintillators) and composite material scintillators (e.g., ZnS­(Ag): PP).^22,23^ Plastic scintillators suffer from sensitivity to background interference, whereas inorganic scintillators suffer from inefficient neutron detection. The combined use of the two is constrained by system complexity and challenges in signal discrimination accuracy. Researchers have explored hybrid two-dimensional (2D) lead (Pb)-based perovskites to address these limitations and develop high-performance scintillators with the molecular-level coupling of organic and inorganic components. Notably, Mn-(C_18_H_37_NH_3_)_2_PbBr_4_ has been reported as a large-area, self-supporting fast neutron scintillator plate, achieving an impressive spatial resolution of 0.5 lp/mm.^24^ The radioluminescence (RL) in this material originates from manganese (Mn^2+^) ions. Another perovskite scintillator, (C_4_H_9_NH_3_)_2_PbBr_4_, has demonstrated high light yield for fast neutrons and 22,000 photons/MeV for X-rays.^25^ More importantly, this material enables energy-selective fast neutron and X-ray imaging, achieving high spatial resolutions of about 1 and 17.3 lp/mm, respectively.^25^ Further studies on (C_4_H_9_NH_3_)_2_PbBr_4_ single crystals have revealed a linear energy response to fast neutrons, making them highly promising for advanced imaging applications.^20^ Additionally, hybrid perovskites, such as (C_8_H_12_N)_2_PbBr_3.8_Cl_0.2_, have emerged as promising candidates for mixed-field radiation detection, with an exceptional light yield of 41,000 photons/MeV and outstanding linearity in response to fast neutrons and gamma-rays, surpassing conventional scintillators.^21^ High-quality, inch-sized (PEA)_2_PbBr_4_ single crystals have also been explored as synergistic scintillators for high-energy radiation and charged particle detection, delivering a high light yield (38,600 photons/MeV) and exceptional spatial resolutions of 23.2 lp/mm (modulation transfer function [MTF] = 0.2) for X-rays and 2.00 lp/mm for fast neutrons, outperforming previous perovskite-based scintillators.^13^ Despite their excellent performance, these lead-containing materials raise significant environmental and biological concerns due to the unavoidable use of lead-containing raw materials and the treatment of lead-containing waste.

In contrast, research on X-ray imaging scintillators has advanced more extensively, particularly in the development of low-dimensional metal halides, flexible scintillator screens, and luminescent free-radical scintillators.^26–29^ Among these, organic–inorganic hybrid copper­(I) halides have attracted considerable attention because of their facile synthesis, biocompatibility, structural diversity, and high X-ray scintillation efficiency. For example, Xia et al. reported a zero-dimensional (C_8_H_20_N)_2_Cu_2_Br_4_ with a PLQY of 99.7% and a light yield of ∼91,300 photons/MeV.^30^ The excellent X-ray scintillation performance of copper­(I) halides mainly originates from their inorganic CuX framework, which provides efficient X-ray absorption. At the same time, the organic component in hybrid copper­(I) halides can interact with fast neutrons and enable light emission under neutron irradiation, making this class of materials suitable for fast neutron imaging, as well. Therefore, hybrid copper­(I) halide scintillators represent promising candidates for X-ray and fast neutron dual-mode imaging. To the best of our knowledge, however, no such report has been published.

This study develops a high-performance, Pb-free multifunctional scintillator PPDCuI (molecular formula of C_24_H_40_N_8_P_6_O_12_Cu_4_I_6_) hybrid copper­(I) iodide. The material emits yellow light when excited by ultraviolet (UV) light at room temperature, with a photoluminescence quantum yield (PLQY) of up to 97.65%. The variable-temperature PL and lifetime analysis reveal that the luminescence mechanism of PPDCuI is thermally activated delayed fluorescence (TADF). This unique characteristic endows the material with significant advantages for optoelectronic applications. Notably, fast neutron imaging was realized using a wafer of PPDCuI, achieving a high spatial resolution of 1.47 lp/mm. The organic component of the material serves as a neutron moderator, whereas the inorganic core converts the residual kinetic energy of neutrons into emitted photons, facilitating effective neutron detection. Furthermore, this work explores the potential of PPDCuI for X-ray imaging. The material demonstrates a high light yield of about 42,000 photons/MeV, and the polymer film scintillator fabricated with poly­(methyl methacrylate) (PMMA) and PPDCuI achieved an exceptional spatial resolution of 25.8 lp/mm under X-ray irradiation. This study confirms the first successful realization of fast neutron imaging using hybrid copper­(I) iodide with TADF characteristics while highlighting the significant potential for high-resolution X-ray imaging.

## Results and Discussion

The single crystal of the material PPDCuI was synthesized by improving the reported method (the single-crystal data are shown in Table S2, CCDC No. 2401669), and the powder was prepared using the solution coprecipitation approach developed in this study.^31^ Typically, synthesizing a hybrid copper­(I) halide does not require adding antioxidants to protect Cu^+^ ions. However, in the synthetic system, the absence of hypophosphorus acid (H_3_PO_2_) causes rapid oxidation in Cu^+^ ions via atmospheric oxygen (O_2_). Introducing H_3_PO_2_ prevents Cu^+^ oxidation and becomes an integral part of the material composition. Moreover, *p*-phenylenediamine (PPD) undergoes protonation, forming PPD cations. The three H atoms on the nitrogen (N) atom of PPD establish H bonds (H–O) with three PO_2_ groups, forming a layered structure. This structural arrangement isolates the typically independent amine ligands from the inorganic [Cu_4_I_6_]^2–^ ionic modules, as illustrated in Figure 1a.

**Figure 1:**
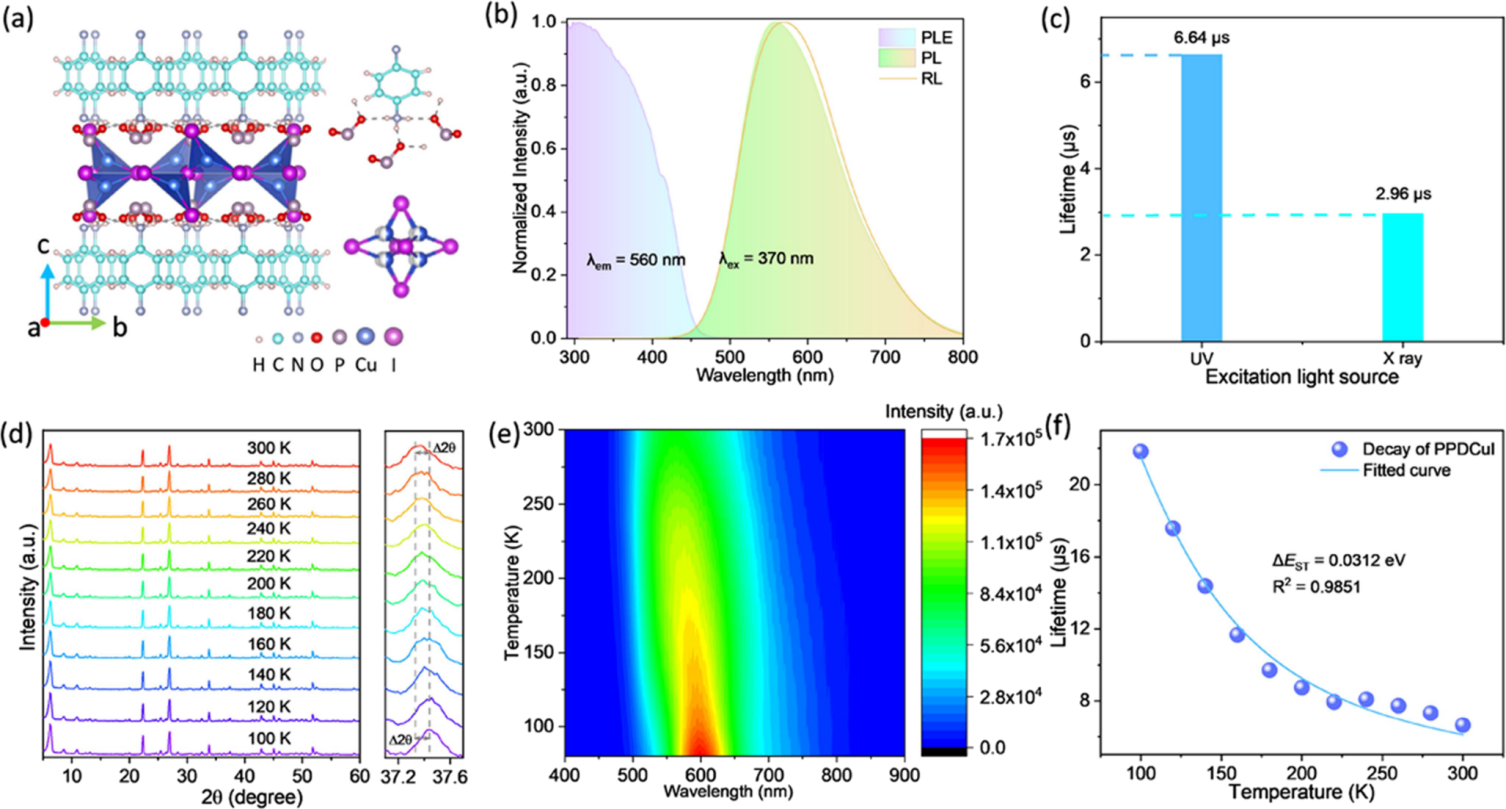
(a) Crystal structure of PPDCuI (molecular formula C_24_H_40_N_8_P_6_O_12_Cu_4_I_6_; PPD = *p*-phenylenediamine) viewed along the *a*-axis (left). Structure of [PPDH]^2+^ and [PO_2_]^−^ (upper right). Detailed view of [Cu_4_I_6_]^2–^ aggregation (low right). (b) Normalized photoluminescence (PL) excitation (PLE), PL, and radioluminescence (RL) spectra of PPDCuI. (c) PL (left) and RL (right) lifetimes of PPDCuI. (d) Temperature-dependent powder X-ray diffraction (XRD) of PPDCuI (*T* = 100 to 300 K). (e) Temperature-dependent PL (TDPL) of PPDCuI (*T* = 100 to 300 K) excited at 370 nm. (f) Lifetimes of TDPL monitored at 550 nm (*T* = 100 to 300 K) and the fitted curve of the Boltzmann equation.

Furthermore, Figure S1 compares the experimental powder X-ray diffraction (XRD) pattern with the simulated XRD pattern derived from the single-crystal structure, demonstrating an excellent match and confirming the high phase purity of the synthesized material. The single-crystal structure analysis reveals that the material crystallizes in the tetragonal system with space group *P*4/*m* and unit cell parameters *a* = *b* = 13.814(2) Å, *c* = 13.146(4) Å, and α = β = γ = 90°.

In order to study the oxidation state of P in PPDCuI, we first conducted a solid-state nuclear magnetic resonance (ssNMR) test on the sample. The results show that there was only one chemical environment of P in our as-synthesized sample (Figure S2). Additionally, an X-ray photoelectron spectroscopy (XPS) analysis was conducted to confirm the oxidation state of phosphorus (P) in the material (Figure S3). The chemical state of P in PPDCuI is similar to that of P in the standard sample Na_2_HPO_3_, indicating that P in both of them has the same oxidation state. An electron paramagnetic resonance (EPR) analysis was performed to further confirm the oxidation state of phosphorus (P) in the material (Figure S4). The EPR spectrum displayed two distinct signals at *g* = 2.00 and 1.93. The higher oxidation state of P corresponds to a lower g-value in its EPR signal. Based on this trend, the observed signals suggest the presence of higher-valence P, ruling out the possibility of +1 valence P in the [H_2_PO_2_]^−^ group. Consequently, the material is confirmed to contain PO_2_ units. Specifically, the *g* = 2.00 signal indicates the partial localization of unpaired electrons on O, whereas the *g* = 1.93 signal suggests the primary localization of unpaired electrons on the 3p orbital of P. These findings confirm that P in the hybrid adopts a + 3 oxidation state rather than the +1 state characteristic of H_3_PO_2_, aligning with the EPR results.

The spectroscopy properties of PPDH_2_CuX (X = Cl, Br) and PPDCuI were systematically investigated (Figures 1 and Figures S5–S18). Figure 1b presents the PLE spectrum monitored with the emission band at 560 nm, revealing a wide excitation band (from 250 to 450 nm) with peaks at ∼300 nm; it covers a similar spectrum range with the UV–visible absorption spectrum (Figures S5). Upon excitation at 370 nm, PPDCuI presents a bright emission with a peak at 560 nm and a full width at half-maximum (fwhm) value of 143 nm. The PLQY of the PPDCuI can achieve 97.65% at room temperature (Figure S8c). These spectral characteristics indicate the presence of inorganic module-based luminescence. The detailed size of the model is shown in Figure S19a. Density functional theory (DFT) calculations show that the valence band maximum (VBM) is mainly contributed by Cu and I atoms, while the conduction band minimum (CBM) is primarily composed of Cu-derived orbitals. The inset partial charge densities in the energy window of −1 to 0 eV are assigned to bonding orbitals. Notably, the corresponding charge densities are predominantly distributed on the Cu, indicating significant electronic participation of these species in the bonding states (Figure S19b). Furthermore, the large Huang–Rhys (*S*) factor (*S*_PL_ = 58, *S*_RL_ = 169) obtained from the variable-temperature PL and variable-temperature RL also indicates the strong exciton confinement feature of this material (Figures S13–S17). The temperature-variable RL exhibited a negative thermal quenching characteristic.

Additionally, the excitation-wavelength-dependent emission spectra reveal that the intensity variation of the emission peak with the excitation wavelength follows the same trend as that of the excitation spectrum in this wavelength range (Figure S9). Notably, the shape of the emission spectrum remains unchanged across excitation wavelengths (Figure S9a). Figure 1b presents the normalized PL and RL spectra for a better comparison, and Figure 1c provides their lifetimes. Figures S7c and S12 present the decay and fitted curves of the PL and RL of PPDCuI, respectively. Compared with PL, RL exhibits a slightly wider fwhm and a shorter lifetime, decreasing from 6.64 (PL) to 2.96 μs (RL). Temperature-dependent powder XRD, PL, and PL decay characterizations were performed at 100 to 300 K to elucidate this phenomenon. The temperature-dependent powder XRD patterns shown in Figure 1d reveal that the sample undergoes only regular thermal expansion and contraction within the examined temperature range, without any evidence of phase transitions. This confirms that the temperature-dependent PL (Figure 1e) and PL decays are not influenced by structural phase changes. As the temperature increases, the PL spectrum broadens (Figures 1e and S13) and blue-shifts, and the triplet lifetime generally decreases (Figure 1f, Figure S18b, and Table S3). The exciton binding energy (*E*_*a*_) of the material was 135.2 meV, and the temperature-dependent PL (TDPL) intensity was fitted by using the Arrhenius eq (Figure S15). Considering that the thermal energy at room temperature is kT ≈ 25.8 meV and *E*_*a*_ > 3 kT, the excitons are expected to be relatively stable under ambient conditions. A sufficiently high exciton binding energy effectively suppresses nonradiative recombination processes, accounting for the high PLQY observed at room temperature.

Based on these results, the observed trends in TDPL and lifetimes are consistent with those in the previous literature.^32,33^ This alignment suggests that the delayed fluorescence originates from thermally activated upconversion, facilitating the transition from the lowest triplet state (T_1_) to the lowest singlet state (S_1_). According to the literature, the relationship between the average decay time (τ_av_) of PL and absolute temperature (*T*) is expressed as follows:^33^

$$\tau_{\mathrm{av}}=\frac{3+e^{-\Delta E_{\mathrm{ST}}/k_{B}\times T}}{3/\tau_{T_{1}}+1/\tau_{S_{1}}\times e^{-\Delta E_{\mathrm{ST}}/k_{B}\times T}}$$

where *k*_B_ denotes the Boltzmann constant, and τ_S_1__ and τ_T_1__ represent the decay times of the S_1_ and T_1_ states, respectively. In addition, Δ*E*_ST_ indicates the energy level difference.

Figure 1f reveals that the experimental τ_av_ data of PPDCuI fit well with the model, yielding fitted values of Δ*E*_ST_ = 0.0312 eV. At low temperatures, excitons predominantly freeze in the triplet state, making T_1_ the dominant emissive state. As the temperature increases, excitons gradually populate the higher-energy S_1_ state. The small energy gap between S_1_ and T_1_ is crucial to facilitate efficient reverse intersystem crossing (RISC), enabling dominant thermally activated delayed fluorescence (TADF) at ambient temperature. The fitting to the average lifetimes and the results proved the TADF mechanism of the material. The TADF materials display high theoretical quantum efficiency due to the ability to convert transition-forbidden triplet excitons into radiative singlet excitons, making them promising for optoelectronic device applications.

The determined TADF mechanism can explain the spectral characteristic changes caused by the temperature change and X-ray excitation. Under UV excitation, with the singlet and triplet energy-level broadening with the temperature increase, phonon-assisted radiative transitions from higher-energy singlet states increase, causing PL broadening. More significantly, excitons in the triplet excited state are increasingly prone to RISC to the singlet state with phonon assistance, where radiative recombination occurs, resulting in the observed PL blue shift and shortened lifetime. Meanwhile, stronger phonon interactions caused wider emission energy levels and more nonradiative recombination channels, which induce the radiative emission broadening and a shorter lifetime. For temperature-dependent RL (X-ray excitation), the blue shift and emission peak broadening phenomena are consistent with those of temperature-dependent PL, but the trend of RL intensity variation differs slightly from that of temperature-dependent PL. Under X-ray excitation, the RL intensity first increases and then decreases with increasing temperature. This is mainly because the ionization of the material under X-ray excitation generates a larger population of triplet excitons and stronger electron–phonon interaction (*S*_PL_ = 58, *S*_RL_ = 169), leading to temperature-dependent RL behavior different from that observed under photoexcitation.^34^

As mentioned in the introduction, organic–inorganic hybrid copper­(I) halides are potential materials for fast neutron detection and imaging. First, we pressed PPDH_2_CuX (X = Cl, Br) and PPDCuI powders into wafers (Figure S20) to compare their luminescence intensity under fast neutron irradiation. The results show that the RL intensity under fast neutron irradiation gradually increased from Cl to Br to I (Figure S21). Given the high luminescence efficiency and TADF luminescence mechanism of PPDCuI, we selected PPDCuI for a detailed investigation and dual-mode X-ray/fast neutron imaging studies.

In PPDCuI, the scintillation process for fast neutrons proceeds through a distinct mechanism: upon collision with an H nucleus, a fast neutron generates a recoil proton and a scattered neutron. Having acquired substantial kinetic energy, the recoil proton acts as a high-energy particle and interacts with the electrons of heavy atoms in the scintillator, exciting them to higher-energy states without ionization. These excited electrons are likely to occupy singlet or triplet states. The thermally assisted RISC process, facilitated by phonon interactions, enables some triplet excitons to upconvert to singlet states. The exciton population on singlet and triplet states, which subsequently relaxes to the ground state, results in photon emission, producing fast neutron-induced RL. The schematic diagram of the fast neutron excitation of PPDCuI is shown in Figure 2a.

**Figure 2:**
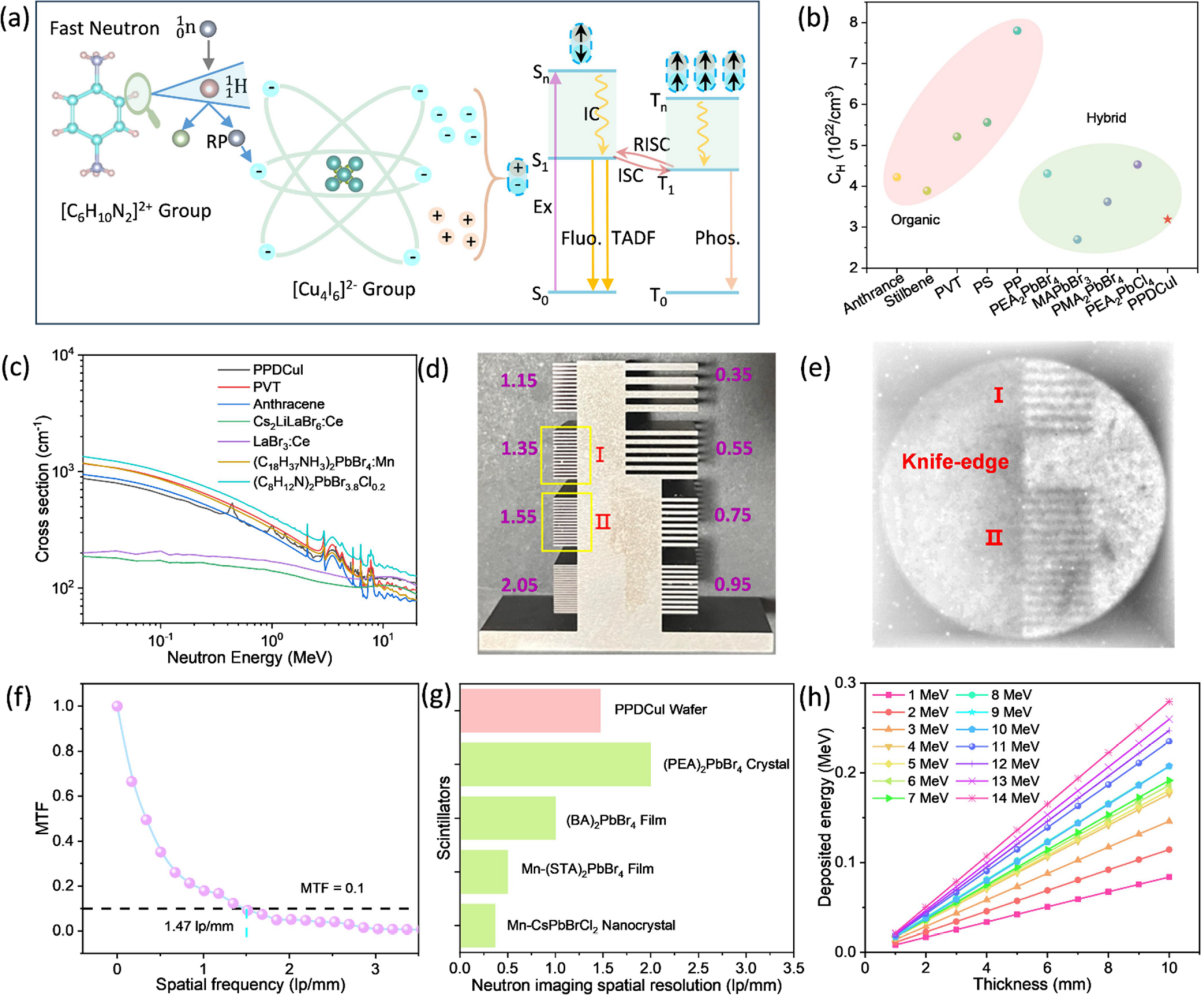
(a) Schematic diagram of the fast neutron excitation of PPDCuI to produce radioluminescence (RL). (b) Hydrogen atom concentration of neutron scintillators. (c) Macroscopic cross sections of scintillators toward neutrons. (d) Photograph of a tungsten line-pair standard test pattern plate. Spatial resolutions corresponding to regions I and II are 1.35 and 1.55 lp/mm, respectively. (e) Fast neutron image of the PPDCuI wafer for a standard test pattern plate. (f) Modulation transfer function (MTF) curve for the PPDCuI wafer. (g) Recently reported spatial resolutions of neutron scintillators in hybrid materials.^5,13,24,25^ (h) Deposited energies of different neutron energies (1–14 MeV) versus thicknesses (1–10 mm).

The foregoing analysis underscores the critical dependence of a material’s fast neutron sensitivity on its H atom concentration. Systematic calculations of H concentrations were performed to elucidate this structure–property relationship for a series of representative neutron scintillators. As summarized in Figure 2b and tabulated in Table S4, the H atom concentration of the hybrid scintillator PPDCuI (3.19 × 10^22^ cm^–3^) exceeds that of the perovskite-based MAPbBr_3_ (2.70 × 10^22^ cm^–3^) but remains lower than the layered hybrid perovskite scintillator PEA_2_PbBr_4_ (4.31 × 10^22^ cm^–3^) and commercial organic counterparts, such as anthracene (4.22 × 10^22^ cm^–3^) and stilbene (3.89 × 10^22^ cm^–3^).^21,23,35^ Figure 2c shows the cross sections of PPDCuI and some other scintillators toward neutrons. The cross section of PPDCuI is much larger than that of inorganic scintillators such as Cs_2_LiLaBr_6_: Ce and LaBr_3_: Ce when the neutron energy is lower than 10 MeV. When the neutron energy is higher than 1 MeV, the cross section of PPDCuI is greater than that of anthracene and approaches that of PVT. And as the neutron energy increases, the cross section of PPDCuI still maintains an increasing trend compared to that of anthracene and PVT. Notably, although the H atom concentration serves as a primary determinant for neutron interaction cross sections, the radiation-induced recombination dynamics—a critical factor governing scintillation efficiency—must also be rigorously considered to rationalize performance disparities among these materials.

Before fast neutron imaging was conducted, the response of PPDCuI to fast neutrons was assessed by measuring the pulse height spectrum under monoenergetic neutron irradiation (5 and 6 MeV) using a D–D neutron generator at the China Institute of Atomic Energy, Beijing. As illustrated in Figure S22, the pulse height spectrum obtained with the sample differs from the background without neutron irradiation, confirming the robust fast neutron response of PPDCuI. Moreover, the cutoff edge observed in the response spectrum indicates that PPDCuI can differentiate neutrons of various energies, underscoring its suitability for fast neutron imaging applications. Next, fast neutron imaging was performed at the China Spallation Neutron Source, Dongguan, using the back-n-white neutron beamline. The neutron source generates a broad-spectrum neutron beam (0 to 200 MeV) via proton bombardment of a tungsten (W) target, accompanied by a γ-ray background. The time-of-flight method was employed to eliminate γ-ray interference, using a CMOS camera shutter and delay adjustments to select fast neutrons in the 1 to 14 MeV energy range.

Figure 2e shows the fast neutron imaging photo, using a W. standard line-pair test pattern plate (Figure 2d), yielding resolutions of 1.35 and 1.55 lp/mm in regions I and II, respectively. Figure 2f presents the MTF curve obtained by using the edge method, revealing a spatial resolution of about 1.47 lp/mm (at MTF = 0.1) for the PPDCuI wafer, which validates the imaging result.

Figure 2g illustrates the neutron imaging resolutions of some recently reported perovskite-based scintillators. The comparative analysis indicates that the spatial resolution of PPDCuI outperforms most existing perovskite fast neutron scintillators. Furthermore, the straightforward and scalable lamination process for wafer fabrication highlights the potential of the material for large-area, high-performance, fast neutron imaging applications. In addition, we calculated the energy deposition of fast neutrons in the energy range of 1–14 MeV for materials of different thicknesses, and the calculated data were listed in Table S5. As shown in Figure 2h, with the increase of thickness (1–10 mm), the deposition amount of every single energy (1–14 MeV) neutron increases linearly (Figure S23). Moreover, within the range of material thickness 1–10 mm and fast neutron energy 1–14 MeV, the energy deposition of fast neutrons by the PPDCuI material has not yet reached saturation.

This work further explores the X-ray scintillation performance of PPDCuI. First, this material has excellent environmental stability. After being placed in the atmospheric environment for one month (winter in Beijing, China), it can still maintain its original phase structure and can retain more than 90% of the initial irradiated luminescence intensity, as shown in Figures S24 and S25. The X-ray scintillation effect occurs when X-rays interact with a scintillation material such as thallium-doped sodium iodide (NaI­(Tl)), cesium iodide (CsI), or lutetium aluminum garnet (LuAG). This interaction generates high-energy secondary electrons via various processes such as Compton scattering, photoelectric, or electron-pair effects. After the X-ray is absorbed by the inorganic group of PPDCuI, the ground-state electrons of the [Cu_4_I_6_]^2–^ group are excited into the excited state. These excited electrons are transferred from the singlet state to the triplet state by ISC, then back to the singlet state by RISC, and finally transferred back to the ground state by emitting photons, resulting in scintillation. Figure 3a illustrates the general process. Heavy atoms containing more extranuclear electrons than light atoms have a stronger ability to absorb X-rays. Figure 3b exhibits the effective atom numbers (*Z*_eff_) of some organic, hybrid, and inorganic materials (Table S4). The *Z*_eff_ of PPDCuI is less than that of several listed hybrid perovskites and inorganic scintillators but greater than that of the selected organic scintillators.

**Figure 3:**
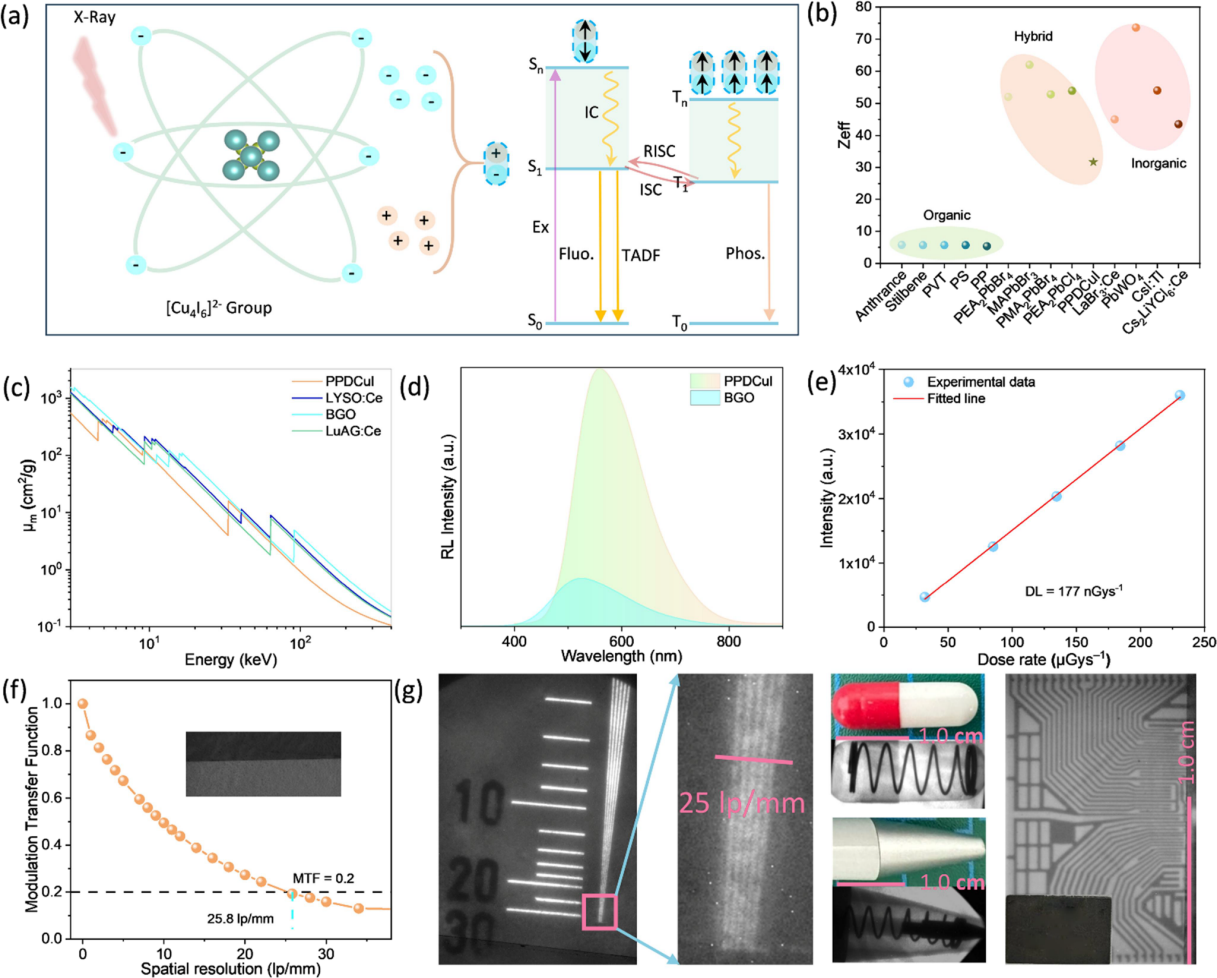
(a) Schematic diagram of the X-ray excitation of PPDCuI to produce radioluminescence (RL). (b) Effective atomic numbers of some representative scintillators. (c) Calculated X-ray mass absorption coefficients (μ_m_) of PPDCuI, LYSO:Ce, BGO, and LuAG:Ce scintillators as a function of the photon energy from 1 to 200 keV. (d) RL spectra of BGO and PPDCuI. (e) RL intensity of the PPDCuI scintillator as a function of the X-ray dose. (f) Modulation transfer function (MTF) curve for the PPDCuI@PMMA scintillator. The inset shows the X-ray edge image used for the MTF calculation. (g) X-ray image of a standard line-pair card and a partial enlarged detail of the line-pair card imaging. Photographs and X-ray images of a spring encapsulated in a capsule, a ballpoint pen, and a chip. The scale bar is 1.0 cm.

Efficient X-ray attenuation is a fundamental requirement for high-performance scintillators. Figure 3c plots the X-ray μ_m_ of PPDCuI and reference materials such as LYSO:Ce, BGO, and LuAG:Ce. From the entire energy range, the X-ray μ_m_ of PPDCuI is slightly lower than those of the three commercially available inorganic scintillators. Moreover, X-rays are more sensitive to elements with high atomic numbers; thus, it is beneficial for hybrid materials to obtain X-ray attenuation coefficients close to those of inorganic scintillators. Notably, a 1.5-μm-thick PPDCuI sample can absorb over 90% of X-ray photons at 30 keV (Figure S26), confirming its effective X-ray attenuation ability.

A high light yield is also critical for achieving high-resolution X-ray imaging. We measured the RL spectra of PPDCuI alongside commercial scintillators (LYSO, BGO, and CsI:Tl) under identical experimental conditions, as shown in Figure S27. The commercial scintillator BGO was used as a standard reference. The light yield of the BGO scintillator is about 10,000 photons/MeV.^36^ By integrating the X-ray-induced RL spectra of PPDCuI and BGO and comparing the results (Figure 3d), the light yield of the PPDCuI scintillator was indirectly determined at about 42,000 photons/MeV, higher than the literature-reported.^37^ This value is comparable to those of some excellent all-inorganic perovskite scintillators and is higher than the values for most organic plastic and liquid scintillators for X-ray detection. As expected, the high Z-element content in the inorganic part of the PPDCuI framework provided a large X-ray absorption cross section, contributing significantly to this high light yield. The high light yield of PPDCuI also benefits from the TADF process. Similar to neutron RL, the phonons produced via the X-ray irradiation of materials are also applied to assist the RISC process, enhancing radiative recombination rather than inducing nonradiative recombination channels.

We assessed the performance of the flexible scintillator using PPDCuI@PMMA film with a thickness of 120 μm, which was fabricated by integrating 100 mg of PPDCuI powder with 200 mg of PMMA. Such a thickness can ensure the complete absorption of X-rays. The SEM images and EDS mapping results show that the particle distribution is uniform and the film surface is smooth and flat (Figures S28 and S29). The detection limit is another critical parameter for evaluating scintillator performance because it determines the minimum detectable X-ray dose. Figure 3e illustrates the strong linear correlation between the X-ray dose rate and RL intensity of the PPDCuI@PMMA film scintillator when the X-ray dose rate ranges from 31.91 to 230.80 μGyair s^–1^, and Figure S30 presents the RL spectra. A low detection limit of 177 nGyair s^–1^ was determined for a signal-to-noise ratio of 3, 30-fold lower than the dose rate required for typical medical X-ray diagnoses (5.5 μGyair s^–1^).^38^ Thus, the radiation dose exposure of the patient would be considerably reduced when the system is employed in medical radiography. The irradiation stability of these materials is notable, maintaining 95% of their initial RL intensity even after 1000 s of continuous X-ray exposure (Figure S31). Combined with the high light yield and excellent irradiation stability, these values confirm that the PPDCuI@PMMA film scintillator is a promising candidate for commercial scintillator applications.

This work characterizes the X-ray imaging performance of the film scintillator by using a custom-built X-ray imaging system. The spatial resolution was first evaluated by measuring the MTF curve using the edge spread function method (Figure 3f). The PPDCuI@PMMA film scintillator demonstrated a spatial resolution of 25.8 lp/mm at an MTF of 0.2. A standard test pattern board further verified the high-resolution X-ray imaging capability (Figure 3g). A spatial resolution of about 25 lp/mm was achieved, aligning well with the results from the MTF method. Such X-ray imaging performance has surpassed that of many reported similar materials (Table S6). Then, a capsule-wrapped spring and an opaque ballpoint tip were used as imaging objects. The X-ray images revealed their internal spring with sufficient contrast and sharpness. Next, we further assessed a microchip as an imaging object. The details of the internal circuitry in the microchip were resolved without blurring. This result confirms the potential of the PPDCuI@PMMA film scintillator for X-ray imaging applications.

## Conclusions

In conclusion, this work presents a multifunctional, eco-friendly hybrid material, PPDCuI, which significantly advances both X-ray and fast neutron imaging. More specifically, this well-designed scintillator incorporates an H-rich organic framework, enabling effective, fast neutron absorption, whereas its inorganic component facilitates the rapid conversion of fast neutrons and X-ray radiation into visible light photons. Fast neutron imaging using PPDCuI wafers achieved an impressive spatial resolution of up to 1.47 lp/mm, whereas the scintillator displayed a remarkable light yield of about 42,000 photons/MeV under X-ray irradiation. X-ray imaging with the PPDCuI@PMMA polymer flexible film also reached a high spatial resolution of about 25.8 lp/mm. The successful application of PPDCuI in fast neutron imaging fosters the development of advanced scintillator materials that combine fast neutron and X-ray imaging capabilities. Our findings not only showcase the first successful realization of fast neutron imaging using a hybrid copper­(I) halide but also reveal new possibilities for advanced imaging applications, including security screening and materials analysis. The environmental friendliness of our proposed material adds a significant dimension to its practical implications, as it addresses the critical need for sustainable alternatives in radiation detection technologies while simultaneously reducing the overall detection costs.

## Supplementary Material



## Notes

Experimental procedures; method; PXRD patterns of PPDCuI; NMR spectroscopy of 31P for PPDCuI; high-resolution XPS spectrum; EPR spectrum; absorption spectrum; the PLE, PL, decay, and PLQY of PPDH_2_CuCl, PPDH_2_CuBr, and PPDCuI; excitation and emission spectra of PPDCuI measured at different wavelengths; emission spectra of PPDCuI measured at different wavelengths at 80 K; RL of PPDH_2_CuCl, PPDH_2_CuBr, and PPDCuI; RL decay of PPDCuI; temperature-dependent PL spectra and normalized; fwhm and integrated PL intensity as a function of temperature for PPDCuI; temperature-dependent RL spectra and normalized; fwhm of RL spectra as a function of temperature for PPDCuI; TDPL decay and lifetimes of PPDCuI singlet and triplet; PDOS of PPDCuI; wafers of PPDCuI; the grayscale images of PPDH_2_CuCl, PPDH_2_CuBr, and PPDCuI under fast neutron irradiation; amplitude spectra of PPDCuI wafer; deposited energies of different thicknesses (1–10 mm) versus neutron energies (1–14 MeV); PXRD of fresh samples and those that have been placed in the air environment for one month; RL spectra of fresh samples and those that have been placed in the air environment for one month; attenuation efficiencies of PPDCuI scintillator as a function of thickness at 30 keV; RL spectra of PPDCuI, BGO, LYSO, and CsI:Tl; SEM images of PPDCuI@PMMA film; EDS mapping of PPDCuI@PMMA film; RL spectra of PPDCuI@PMMA scintillator irradiation under different X-ray doses; the stability of PPDCuI@PMMA scintillator under 30 on/off cycles upon X-ray irradiation; advantages and disadvantages of fast neutron detection vs X-ray detection; reported crystal structure parameters and our result; concentration of hydrogen atoms and effective atomic number of commonly used organic, inorganic and organic–inorganic hybrid scintillators; the imaging performance of reported copper-cluster scintillators (PDF)

## References

[1] Schillinger B., Beaudet A., Fedrigo A., Grazzi F., Kullmer O., Laaß M., Makowska M., Werneburg I., Zanolli C. (2018). Neutron Imaging in Cultural Heritage Research at The
FRM II Reactor of The Heinz Maier-Leibnitz Center. J. Imaging.

[2] Hausladen P. A., Bingham P. R., Neal J. S., Mullens J. A., Mihalczo J. T. (2007). Portable
Fast-Neutron Radiography with The Nuclear Materials Identification
System for Fissile Material Transfers. Nucl.
Instrum. Meth. B.

[3] Zboray R., Adams R., Morgano M., Kis Z. (2019). Qualification
and Development
of Fast Neutron Imaging Scintillator Screens. Nucl. Instrum. Meth. A.

[4] Eberhardt J. E., Rainey S., Stevens R. J., Sowerby B. D., Tickner J. R. (2005). Fast Neutron
Radiography Scanner for The Detection of Contraband in Air Cargo Containers. Appl. Radiat. Isot..

[5] McCall K. M., Sakhatskyi K., Lehmann E., Walfort B., Losko A. S., Montanarella F., Bodnarchuk M. I., Krieg F., Kelestemur Y., Mannes D., Shynkarenko Y., Yakunin S., Kovalenko M. V. (2020). Fast Neutron
Imaging with Semiconductor Nanocrystal Scintillators. ACS Nano.

[6] Kardjilov N., Manke I., Hilger A., Strobl M., Banhart J. (2011). Neutron Imaging
in Materials Science. Mater. Today.

[7] Kiyanagi Y. (2021). Neutron Applications
Developing at Compact Accelerator-Driven Neutron Sources. AAPPS Bull..

[8] An B., Deng Y., Jin Z., Sun S. (2024). Scintillators for Neutron
Detection and Imaging: Advances and Prospects. Adv. Funct. Mater..

[9] Wu W., Lin S., Wang J.-X., Xu Y., He T., Zhou Y., Yuan P., Maity P., Nadinov I., Thomas S., Huang R., De Castro C. S. P., Yin J., Alshareef H. N., Bakr O. M., Mohammed O. F. (2025). Enhanced
X-ray Luminescence in One-Dimensional
Cu–I Coordination Polymers via Ligand Halogen Engineering. Chem..

[10] Wang J.-X., He T., Zhu X., Thomas S., Shao W., Shekhah O., Alshareef H. N., Bakr O. M., Eddaoudi M., Mohammed O. F. (2025). High-Resolution
Dual-Energy X-ray Imaging Enabled by Transparent Thermally Activated
Delayed Fluorescence (TADF) Scintillation Screen. ACS Mater. Lett..

[11] Li J., Hu Q., Xiao J., Yan Z.-G. (2024). High-Stability Double Perovskite
Scintillator for Flexible X-ray Imaging. J.
Colloid Interface Sci..

[12] Tan W., Xiao Y., Zhou C., Jin X., Zhu S., Han M., Tang Z., Zhang Y., Su Z., Chen T., Chen Q., Liang Q., Chen W., Jiang Y. (2024). Transparent
Perovskite Wafers via Nanocrystals Ordered Coalescence Toward Sensitive
and Stable X-Ray Detection and Imaging. Adv.
Funct. Mater..

[13] Yang B., Ouyang X., Zhao X., Su J., Li Y., Zhang S., Ouyang X. (2024). Inch-Sized 2D Perovskite
Single-Crystal
Scintillators for High-Resolution Neutron and X-ray Imaging. InfoMat.

[14] Lehmann E. H., Tremsin A., Grünzweig C., Johnson I., Boillat P., Josic L. (2011). Neutron Imaging —
Detector Options in Progress. Journal of Instrum..

[15] Manke I., Markötter H., Tötzke C., Kardjilov N., Grothausmann R., Dawson M., Hartnig C., Haas S., Thomas D., Hoell A., Genzel C., Banhart J. (2011). Investigation
of Energy-Relevant Materials with Synchrotron X-Rays and Neutrons. Adv. Eng. Mater..

[16] Kardjilov N., Manke I., Woracek R., Hilger A., Banhart J. (2018). Advances in
Neutron Imaging. Mater. Today.

[17] Eberhardt J. E., Rainey S., Stevens R. J., Sowerby B. D., Tickner J. R. (2005). Fast Neutron
Radiography Scanner for The Detection of Contraband in Air Cargo Containers. Appl. radiat. isotopes.

[18] Wang Y., Wang C., Men L., Zhu J., Hu Q., Xiao J., Mohammed O. F. (2025). Rare Earth Double
Perovskites for
Underwater X-ray Imaging Applications. Inorg.
Chem. Front..

[19] Hu Q., Zhang C., Wu X., Liang G., Wang L., Niu X., Wang Z., Si W.-D., Han Y., Huang R., Xiao J., Sun D. (2023). Highly Effective Hybrid Copper­(I)
Iodide Cluster Emitter with Negative Thermal Quenched Phosphorescence
for X-Ray Imaging. Angew. Chem., Int. Ed..

[20] Wan P., Jin T., Gao R., Ouyang X., Feng Z., Niu G., Tang J., Liu L., Ouyang X. (2023). 2D Perovskite Neutron
Scintillators with Nanosecond Time Resolution and Linearity Energy
Response. Adv. Funct. Mater..

[21] Xia M., Niu G., Liu L., Gao R., Jin T., Wan P., Pan W., Zhang X., Xie Z., Teale S., Cai Z., Luo J., Zhao S., Wu H., Chen S., Zheng Z., Xie Q., Ouyang X., Sargent E. H., Tang J. (2022). Organic–Inorganic
Hybrid Perovskite Scintillators for Mixed Field Radiation Detection. InfoMat.

[22] Yang W., Bin T., Heyong H., Bin L., Ke T., Yong S., Wei Y., Chao C. (2013). The Study of Zinc Sulphide Scintillator for Fast Neutron
Radiography. Phys. Procedia.

[23] Brooks F. D., Cilliers W. A., Simpson B. R. S., Smit F. D., Allie M. S., Jones D. T. L., McMurray W. R., Pilcher J. V. (1988). Deuterated Anthracene
Spectrometer for 5–30 MeV Neutrons. Nucl.
Instrum. Meth. A.

[24] Zheng J., Zeng Y., Wang J., Sun C., Tang B., Wu Y., Zhang Y., Yi Y., Wang N., Zhao Y., Zhou S. (2021). Hydrogen-Rich 2D Halide
Perovskite Scintillators for Fast Neutron
Radiography. J. Am. Chem. Soc..

[25] Shao W., Li Q., He T., Zhang Y., Niu M., Wang H., Zhang Z., Zhou Y., Wang J. X., Fan R., Xia X., Bakr O. M., Mohammed O. F., Liang H. (2023). Synergy of Organic
and Inorganic Sites in 2D Perovskite for Fast Neutron and X-Ray Imaging. Adv. Funct. Mater..

[26] Wang Y., Wang C., Men L., Hu Q., Xiao J. (2024). Colloidal
Synthesis of Hollow Double Perovskite Nanocrystals and Their Applications
in X-ray Imaging. Inorg. Chem..

[27] Zhao Y.-N., Yang Q., Yao B.-H., Cao R.-Y., Zhang H., Wei S.-L., Wei D.-H., Li K., Si Y.-B., Zang S.-Q. (2025). Afterglow Copper­(I) Iodine Cluster Scintillator. Angew. Chem., Int. Ed..

[28] Wang J.-X., Shekhah O., Bakr O. M., Eddaoudi M., Mohammed O. F. (2025). Energy
Transfer-Based X-ray Imaging Scintillators. Chem..

[29] Yuan J.-W., Peng Q.-C., Fu J.-C., Yang Q., Gao Z.-Y., Wang Z.-Y., Li K., Zang S.-Q., Tang B. Z. (2023). Highly
Efficient Stable Luminescent Radical-Based X-ray Scintillator. J. Am. Chem. Soc..

[30] Su B., Jin J., Han K., Xia Z. (2023). Ceramic Wafer Scintillation Screen
by Utilizing Near-Unity Blue-Emitting Lead-Free Metal Halide (C_8_H_20_N)_2_Cu_2_Br_4_. Adv. Funct. Mater..

[31] Zhou S., Chen Y., Li K., Liu X., Zhang T., Shen W., Li M., Zhou L., He R. (2023). Photophysical
Studies for Cu­(i)-Based Halides: Broad Excitation Bands and Highly
Efficient Single-Component Warm White-Light-Emitting Diodes. Chem. Sci..

[32] Wang W. F., Xie M. J., Wang P. K., Lu J., Li B. Y., Wang M. S., Wang S. H., Zheng F. K., Guo G. C. (2024). Thermally
Activated Delayed Fluorescence (TADF)-active Coinage-metal Sulfide
Clusters for High-resolution X-ray Imaging. Angew. Chem., Int. Ed..

[33] Peng Q.-C., Si Y.-B., Wang Z.-Y., Dai S.-H., Chen Q.-S., Li K., Zang S.-Q. (2023). Thermally Activated Delayed Fluorescence Coinage Metal
Cluster Scintillator. ACS Cent. Sci..

[34] Ma W., Su Y., Zhang Q., Deng C., Pasquali L., Zhu W., Tian Y., Ran P., Chen Z., Yang G., Liang G., Liu T., Zhu H., Huang P., Zhong H., Wang K., Peng S., Xia J., Liu H., Liu X., Yang M. (2022). Thermally Activated Delayed Fluorescence
(TADF) Organic Molecules for Efficient X-ray Scintillation and Imaging. Nat. Mater..

[35] Haas F. X., McCarthy J. T. (1967). A Stilbene Fast Neutron Spectrometer
for The Study
of The Neutron Spectrum of a Pu Be Source. Nucl.
Instrum. Methods.

[36] Huang R.-W., Song X., Chen S., Yin J., Maity P., Wang J., Shao B., Zhu H., Dong C., Yuan P., Ahmad T., Mohammed O. F., Bakr O. M. (2023). Radioluminescent
Cu–Au Metal Nanoclusters: Synthesis and Self-Assembly for Efficient
X-ray Scintillation and Imaging. J. Am. Chem.
Soc..

[37] Zhao Y., Chen D., Tang H., Liu H., Liu Y., Dang Y., Lin Q. (2024). Cuprous-Based Layered Single-Crystalline
Scintillators for X-ray Detection and Imaging. J. Mater. Chem. C.

[38] Li B., Jin J., Liu X., Yin M., Zhang X., Xia Z., Xu Y. (2024). Multiphase Transformation
in Hybrid Copper­(I)-Based Halides Enable
Improved X-ray Scintillation and Real-Time Imaging. ACS Mater. Lett..

