# Supplementary material for: Thermally Activated Delayed Fluorescence Hybrid Copper(I) Iodide Scintillator for Fast Neutron and X‑ray Imaging
Source: J Am Chem Soc. 2026 Apr 21;148(17):18313–21. doi: 10.1021/jacs.6c03617 (PMC13154183; doi:10.1021/jacs.6c03617)
Supplement: Supplementary file 1 [file ja6c03617_si_001.pdf]

Supporting Information

Thermally Activated Delayed Fluorescence Hybrid Copper(I) Iodide Scintillator for Fast Neutron and X-Ray Imaging

Qingsong Hu,[*,#,1,2,8] Zizhen Bao,[#,3] Hadeer Elsayed,[#,1] Jian-Xin Wang,[#,1] Linyue Liu,[*,4,5] Jiawen Xiao,[*,6] Guangda Niu,[7] Wentao Wu,[1] Tengyue He,[1] Murilo C Faleiros,[1] Bashir E Hasanov,[1] Yan Jiang,[8] Chengkai Zhang,[9] Di Sun,[9] Osman M. Bakr,[1] and Omar F. Mohammed[1*]

[1] Materials Science & Applied Physics Department, Division of Physical Science and Engineering (PSE), King Abdullah University of Science and Technology (KAUST), Thuwal 23955-6900, Saudi Arabia

[2] Hubei Key Laboratory of Low Dimensional Optoelectronic Materials and Devices, Hubei University of Arts and Science, Xiangyang 441053, China

[3] School of Physics and Astronomy, Beijing Normal University, Beijing 100875, P. R. China

[4] School of Nuclear Science and Technology, Xi'an Jiaotong University, Xi'an 710049, China

[5] State Key Laboratory of Intense Pulsed Radiation Simulation and Effect, Northwest Institute of Nuclear Technology, Xi'an 710024, China

[6] Beijing Key Lab of Microstructure and Property of Solids, College of Materials Science and Engineering, Beijing University of Technology, Beijing 100124, China

[7] Wuhan National Laboratory for Optoelectronics and School of Optical and Electronic Information, Optical Valley Laboratory, Huazhong University of Science and Technology, Wuhan 430074, China

[8] Experimental Center for Advanced Materials, School of Materials Science and Engineering, Beijing Institute of Technology, Beijing 100081, China

[9] School of Chemistry and Chemical Engineering, State Key Laboratory of Crystal Materials, Shandong University, Ji'nan 250100, People's Republic of China.

Corresponding author: hqs@hbuas.edu.cn; liulinyue@nint.ac.cn; xiaojw@bjut.edu.cn; omar.abdelsaboor@kaust.edu.sa

# Qingsong Hu, Zizhen Bao, Hadeer Elsayed, and Jian-Xin Wang contributed equally to this work.

Experimental Section

Chemicals

p-Phenylenediamine (PPD) ($C_6H_8N_2$, 97%, Thermo Scientific), Copper(I) iodide (CuI, 99.9%, Aldrich), Copper(I) bromide (CuBr, 99.9%, Aldrich), Cuprous chloride (CuCl, 99.9%, Aldrich), Hypo phosphorous acid (H_3PO_2, 50% W/W aq. Soln., Thermo Scientific), Ethanol (C_2H_6O, 96%, VWR), Acetonitrile (C_2H_3N, 99.7%, VWR), Chloroform ($CHCl_3$, High-performance liquid chromatography (HPLC)-grade solvents Fisher scientific), polymethyl methacrylate (PMMA, Sigma Aldrich). All reagents used in the experiments were used as received without further purification.

Synthesis

PPDCuI Single Crystal Preparation Method

1 mmol of p-phenylenediamine (PPD) and 0.5 mmol of copper(I) iodide (CuI) were mixed in a Teflon-lined stainless-steel autoclave. To this mixture, 5 mL of acetonitrile (ACN) and 1.5 mL of hypophosphorous acid (H_3PO_2) were added under stirring to ensure homogeneity. The autoclave was then sealed and subjected to hydrothermal conditions at 120°C for 10 hours. After the reaction, the system was cooled down to room temperature naturally. The resulting PPDCuI single crystals were collected and dried under vacuum for further characterization.

PPDCuX (X = Cl, Br, I) Powder Preparation Method

To prepare PPDCuX powder, 1 mmol of PPD (p-Phenylenediamine) was weighed and dissolved in 10 mL of ethanol under stirring until a clear solution was obtained. Separately, 1 mmol of CuX (X = Cl, Br, I) was dissolved in 20 mL of acetonitrile, followed by the addition of 0.5 mL of hypo phosphorous acid (H_3PO_2). The copper iodide solution was added dropwise into the PPD solution, a white precipitate was produced immediately after the two solutions contact. Then centrifuged the mixture and discarded the supernatant. The obtained powder was thoroughly washed twice with 20 mL of ethanol at 8000 RPM for five minutes to remove residual impurities, then vacuum-dried overnight at room temperature.

PPDCuI@PMMA Scintillation Screen Preparation Method

To prepare the PPDCuI@PMMA scintillation screen, 200 mg of PMMA (polymethyl methacrylate) was dissolved in 2 mL of chloroform under continuous stirring until completely dissolved. Separately, 100 mg of PPDCuI powder was finely ground and added to the PMMA solution. The mixture was stirred continuously for 24 hours to ensure uniform dispersion of the PPDCuI particles within the PMMA matrix. The resulting ink was drop-cast onto a clean glass substrate with dimensions of 2.5 cm × 2.5 cm. Once dried, the film was carefully peeled off from the glass substrate to obtain the freestanding PPDCuI@PMMA scintillation screen.

Instrumentation and Characterizations

Phase, Morphology, and Chemical Composition:

X-Ray powder diffraction (PXRD) patterns were collected at room temperature using a Bruker D8 advance diffractometer with Cu Kα radiation (λ = 1.5406 Å) at voltage = 40 kV, current = 40 mA. Phase purity analysis by comparing with the standard PDF card. SCXRD measurements were conducted on a Bruker D8 Venture diffractometer with a SMART APEX2 area detector (Mo Kα, λ = 0.71073 Å). X-ray photoelectron spectroscopy (XPS) was conducted on a Kratos Analytical AMICUS spectrometer, which features a monochromatic Al Kα X-ray source (hv = 1486.6 eV) operating at 75 W under a vacuum of 1×10^{-8} mbar. The morphological characterizations of PPDCuI@PMMA film was carried out by a Quattro S environmental scanning electron microscope (ESEM) (Thermo Fisher Scientific, FEI, USA) and a Zeiss Merlin SEM. The acquisition of the spectra was conducted using the single-pulse (onepulse) excitation sequence from the Bruker pulse program library. A radiofrequency (RF) excitation pulse was employed at a power level of 100 W, with a pulse duration of 5 μs (π/2 pulse). The recorded spectra featured a spectral width of 385 ppm and a time-domain size of 4096 data points.

Computational method

DFT calculations were performed using the Vienna Ab initio Simulation Package (VASP).[1, 2]. The projector augmented wave (PAW) method was used to describe the ion-electron interactions.[3, 4] The generalized-gradient approximation (GGA) with the Perdew-Burke-Ernzerhof (PBE) functional[5, 6] was used as the exchange-correction functional. DFT-D3 Grimme's scheme method[7] for van der Waals correction was adopted for the calculations. The kinetic energy cutoff for plane wave expansions was set to 450 eV. The reciprocal space was sampled using the Gamma center 3×3×3 k-point mesh. All the geometric structures were optimized until the maximal components of forces converged to within 1×10^{-3} eV·Å^{-1}.

Photoluminescence Performance:

The room-temperature steady-state photoluminescence and excitation photoluminescence (PL and PLE) spectra were measured using a Horiba Fluoromax-4 fluorescence spectrophotometer to determine the emission and excitation wavelength. Photoluminescence quantum yield (PLQY), temperature dependent photoluminescence, and time-resolved photoluminescence (TRPL) decay curves were measured using the steady- transient- state fluorescence spectrometer FS5 (Edinburgh Instruments), and the lifetime was calculated according to exponential fitting.

Huang−Rhys factor (S):

The electron−phonon interactions can be revealed from the different full width at half maximum (FWHM) values under variant temperatures by the following equations:

$$\mathrm{FWHM} = 2.36\sqrt{S}\hbar\omega_{phonon}\sqrt{coth\frac{\hbar\omega_{phonon}}{2k_BT}}$$

where S is the Huang−Rhys factor, $\hbar$ is the reduced Planck constant, ω_{phonon} is the phonon frequency, k_B is the Boltzmann constant, T is the temperature.

X-ray Radioluminescence (RL) Properties and Detection Limit:

RL spectra were obtained by fluorescence spectrometer FS5 Edinburgh Instruments with

the XS1-CW external chamber with the CW X-ray source. The detection limit is evaluated by quantifying the optical power excited by X-ray photons. The sample is placed on a precision silicon-based optical power meter (model 843-R, Newport) with copper foil inserted between them to minimize the influence of transmitted X-ray photons on the power meter's response. Signal-to-noise ratio method was employed to determine the detection limit. Fixing the voltage and increasing the current to alter the X-ray dose, the signal-to-noise ratio correlates with the optical power meter's readings. When the reading of optical power meter is 0.01 nw, it is marked as 1 signal-to-noise ratio, and so on. Precise measurement was taken of the distance between the X-ray tube and the power meter. Subsequently, the optical power is measured at various X-ray dose rates. Then, the sample and power meter are taken out, the dosimeter (Accu-Gold Diagnostic System, Radcal) is positioned at the same location as the sample, and the dose rate for the corresponding voltage and current are recorded. At the signal-to-noise ratio of 3, the detection limit of the scintillator is obtained.

Determination of The Light Yield:

We estimated the light yields by using the commercial scintillators BGO. After grinding the single crystal, weighed 0.3 g powder and press it into round wafer with a diameter of about 12 mm and a thickness of 0.5 mm to ensure full absorption of X-rays. The normalized RL spectrum of each reference and sample was calculated using

$$LY(photons/MeV) = \frac{channel_{BGO}}{channel_{sp}} \cdot \frac{gain_{sp}}{gain_{BGO}} \cdot \frac{1}{EWQE} \cdot \frac{1}{0.662}$$

$$\frac{LY_{sample}}{LY_{reference}} = \frac{\eta_{reference} \int I_{sample}\,(\lambda)d\lambda\ \times S_{reference}}{\eta_{sample} \int I_{reference}\,(\lambda)d\lambda\ \times S_{samlpe}}$$

Where, channel represents the number of channels where the full-energy peak of the sample located. Gain represents the gain used in the test, and the gain value can only be determined by adjusting the position to make the full-energy peak appear completely. EWQE stands for emission-weighted quantum Efficiency. η is the percentage of X-ray deposition energy of the scintillator. I is the RL intensity at different λ (wavelength). S is the irradiation area, assuming that the X-ray intensity is uniform across the scintillator film. The

$LY_{reference}$ represents LY_{BGO}.

The absolute light yield of the reference sample had been calibrated by pulse height spectroscopy, and the calibration data were about 10586 photons/MeV for BGO. To reduce the error in the measurement, 10000 photons/MeV was used for BGO.

X-ray Imaging:

X-ray resolution experiments were conducted using a custom-built high-resolution X-ray imaging system. This system includes an X-ray source with a 2 cm aperture (Petrick, Tungsten target) and a CMOS sensor (Basler – 18 μmPro) paired with a telecentric lens (Edmund 0.367x), delivering a resolution of 120 lp/mm at over 20% modulation. High-resolution X-ray images were acquired using a 100 μm thick NCG scintillator screen, operating at 50 kV and 120 μA.

The standard BGO scintillator has a thickness of 0.5 mm. X-ray imaging experiments were conducted using a custom-built system comprising an X-ray source, optical components, and a camera, all enclosed within a lead-shielded box. The spatial resolution of X-ray imaging is typically determined through Modulation Transfer Function (MTF) analysis using the slanted-edge method and further validated with line-pair card images. A Nikon D7100 commercial camera was used to capture images of the X-ray imaging screen. The camera settings were adjusted to ISO 1000, aperture F4, and a shutter speed of 15 seconds for samples excited by X-ray exposure. The X-ray tube operated at 50 kV and 80 μA.

Fast Neutron Imaging:

The pulse height spectra of monoenergetic neutrons at different energies were measured using a D-D neutron source at the China Institute of Atomic Energy. In the experimental setup, the sample was coupled to a photomultiplier tube (PMT, Hamamatsu CR173-Q1), with the opposite end connected to a high-voltage power supply and a preamplifier (Ortec 113). The output of the preamplifier was fed into a spectroscopy amplifier (Ortec 672), and

the resulting pulse height spectrum was recorded using a multichannel analyzer (ORTEC ASPEC-927). Obtained the number of the cut-off channels from the median of shoulder gliding of the pulse spectra.

A fast neutron imaging experiment was conducted in Hall 1 of the Backflow White Neutron (Back-n) beamline at the China Spallation Neutron Source (CSNS) in Dongguan, China. The time-of-flight (TOF) method was employed to regulate the shutter speed and delay time of a CMOS camera, enabling the selection of neutron energies in the range of 1–14 MeV while minimizing the interference of gamma radiation in neutron imaging. During the experiment, when the scintillator was placed in close contact with the tungsten resolution test card, incident neutrons interacted with the scintillator via the (n, p) reaction after attenuation by the tungsten test card, generating a substantial number of recoil protons. These recoil protons further excited the scintillator, producing visible fluorescence. The emitted light was then reflected by a mirror and captured by the CMOS camera to obtain fast neutron imaging photographs. The mirror served to protect the camera from potential damage caused by exposure to high-energy radiation.

Modulation Transfer Function (MTF) Calculation:

The MTF was determined using the slanted-edge method. The edge spread function (ESF) was extracted as the grayscale variation along a specific direction. The line spread function (LSF) was obtained by differentiating the ESF:

$$LSF(x) = \frac{dESF(x)}{dx}$$

Where *x* is the position of pixels. The MTF curve was derived via Fourier transformation of the LSF:

$$MTF(v) = F(LSF(x))$$

Where *v* represents spatial frequency.

Energy Deposition Calculation:

The GEANT4 Monte Carlo program was utilized to systematically simulate the energy deposition distribution under different thickness conditions. In the simulation, the neutron

energy range of 1 to 14 MeV was selected to cover the typical fast neutron energy spectrum. To ensure the statistical reliability of the simulation results, the transport and interaction processes of 5×10^6 incident particles were calculated at each energy point. In terms of geometric modeling, the source type was set as a point source, and scintillation models within the thickness range of 1-10 mm were considered respectively to analyze the influence of thickness on energy deposition efficiency. By statistically analyzing the simulation results, the variation relationship between the average energy deposition of fast neutrons in scintillators of different thicknesses and the incident energy was obtained.

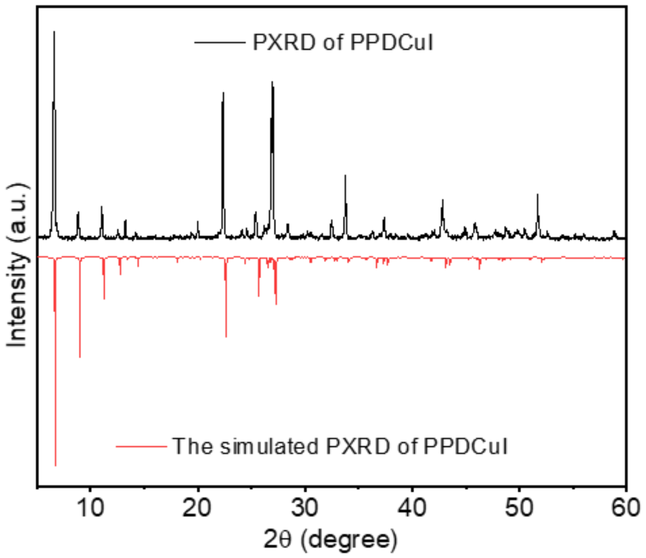

Figure S1. The calculated and experimental powder X-ray diffraction (PXRD) patterns of PPDCuI.

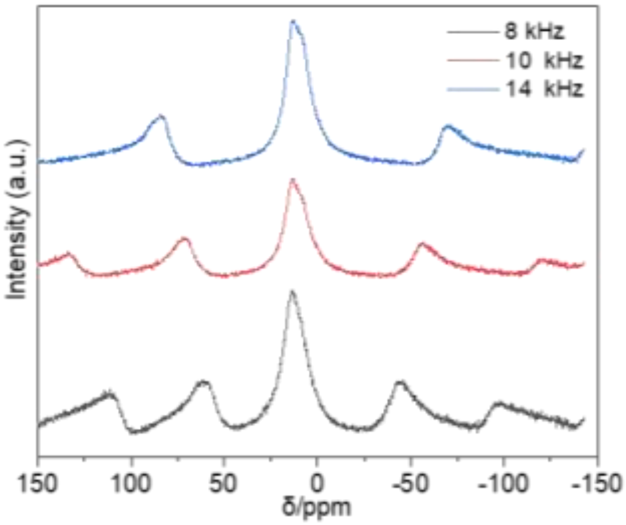

Figure S2. ssNMR spectroscopy of 31P for PPDCuI.

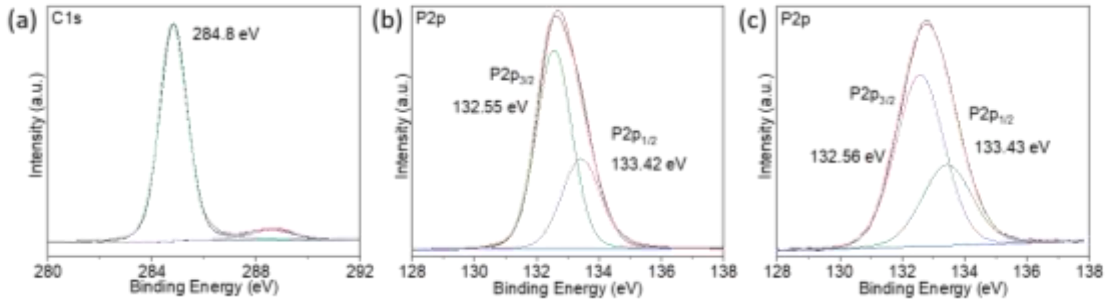

Figure S3. High-resolution XPS spectra of C 1s (a), P 2p for Na_2HPO_3 (b), and P 2p for PPDCuI (c).

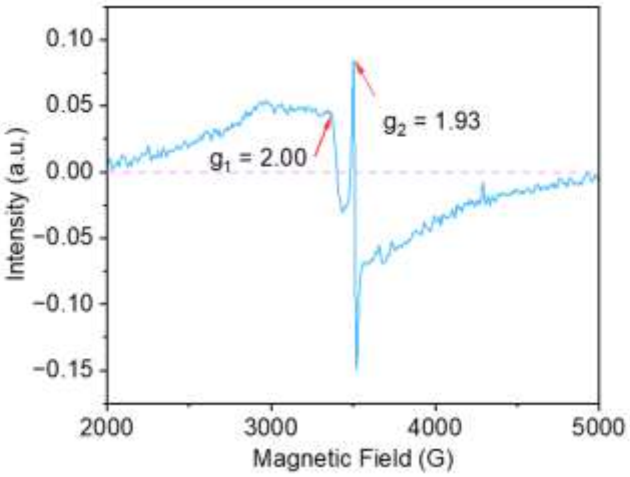

Figure S4. The EPR spectrum of PPDCuI.

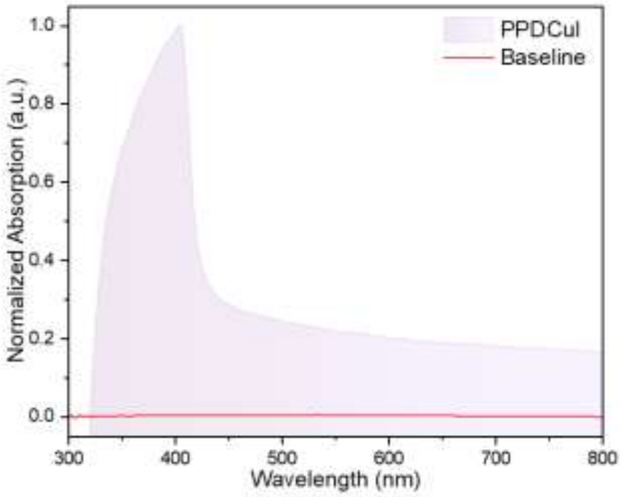

Figure S5. Absorption spectrum of PPDCuI and baseline.

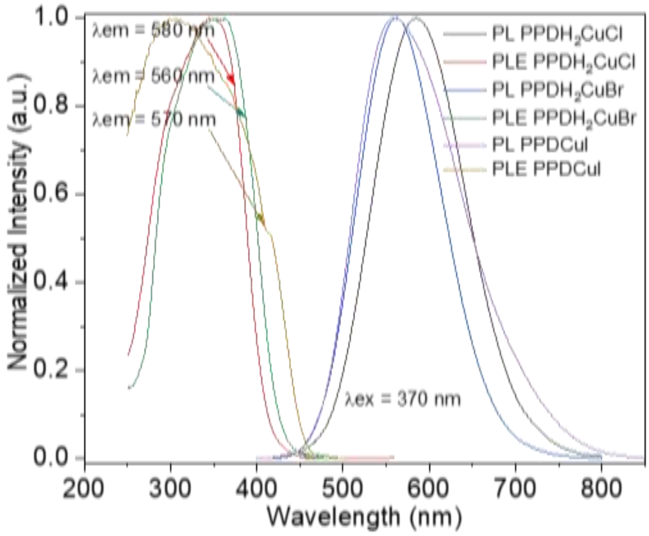

Figure S6. The PLE and PL of $PPDH_2CuCl$, $PPDH_2CuBr$, and PPDCuI.

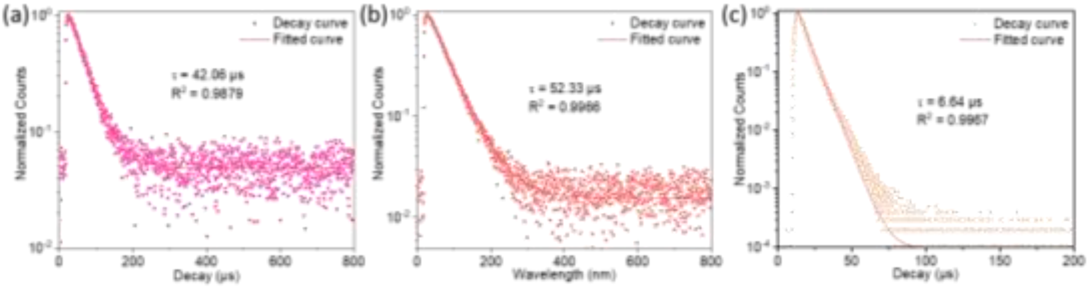

Figure S7. The decay and fitted curves of $PPDH_2CuCl$ (a), $PPDH_2CuBr$ (b), and PPDCuI (c).

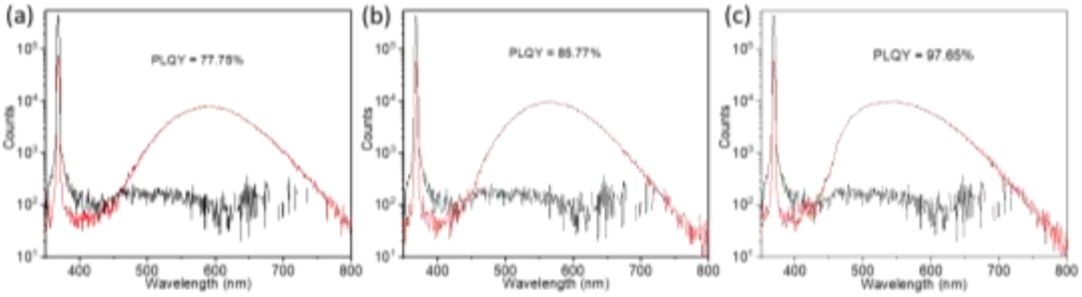

Figure S8. The PLQY of $PPDH_2CuCl$ (a), $PPDH_2CuBr$ (b), and PPDCuI (c).

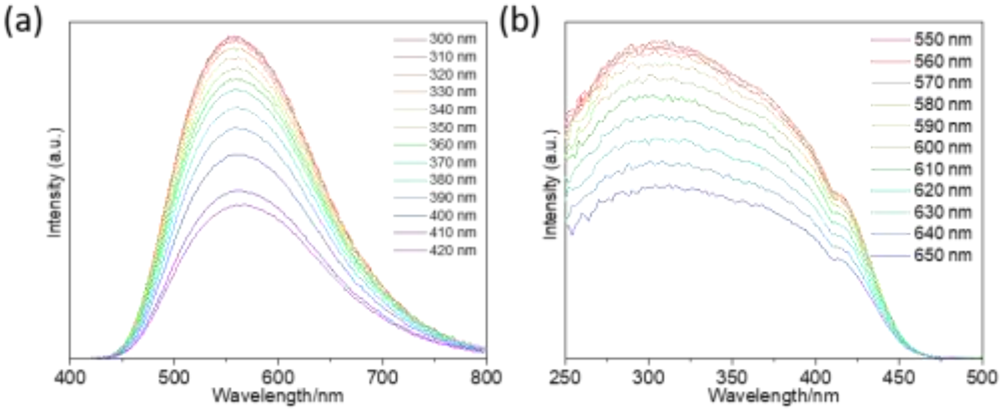

Figure S9. (a) Excitation wavelength dependent photoluminescence (PL) spectra. (b) Emission wavelength dependent photoluminescence excitation (PLE) spectra.

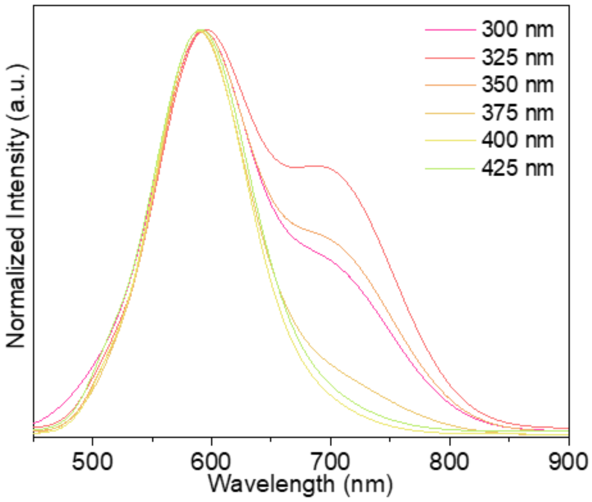

Figure S10. Excitation wavelength-dependent emission spectrum of PPDCuI at 80 K.

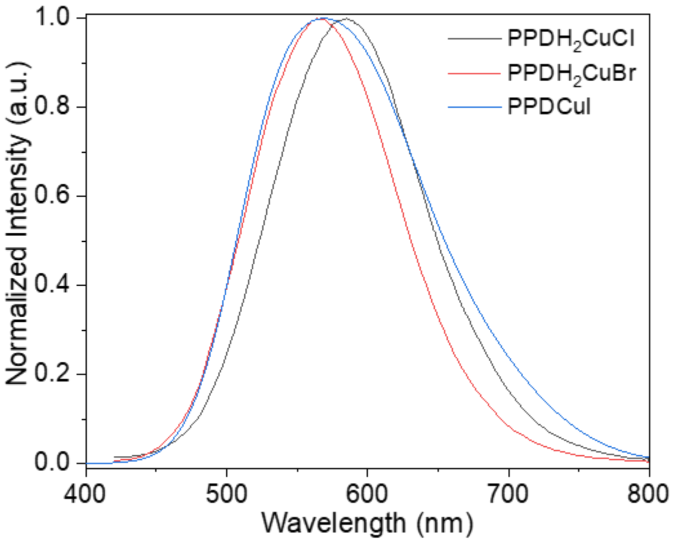

Figure S11. The RL of $PPDH_2CuCl$, $PPDH_2CuBr$, and PPDCuI.

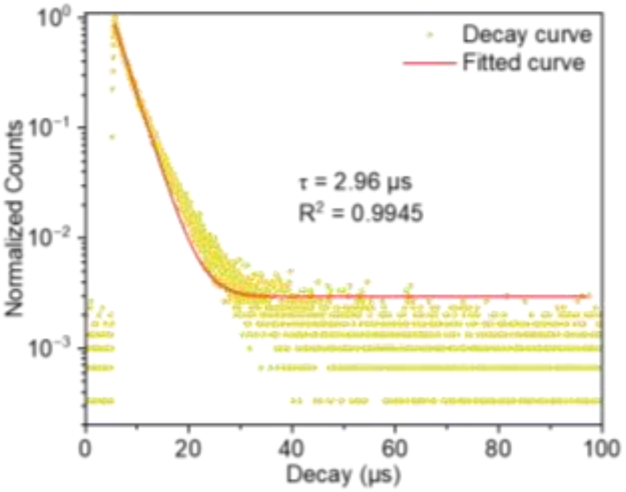

Figure S12. The RL decay and its fitted curves of PPDCuI.

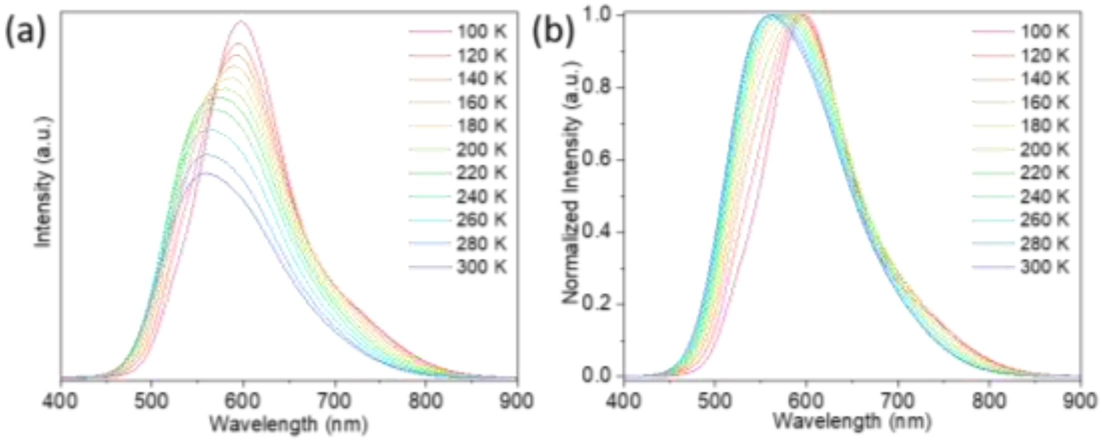

Figure S13. (a) Temperature dependent PL spectra of PPDCuI excited at 370 nm (T = 100 K – 300 K). (b) Normalized Temperature dependent PL spectra of PPDCuI excited at 370 nm (T = 100 K – 300 K).

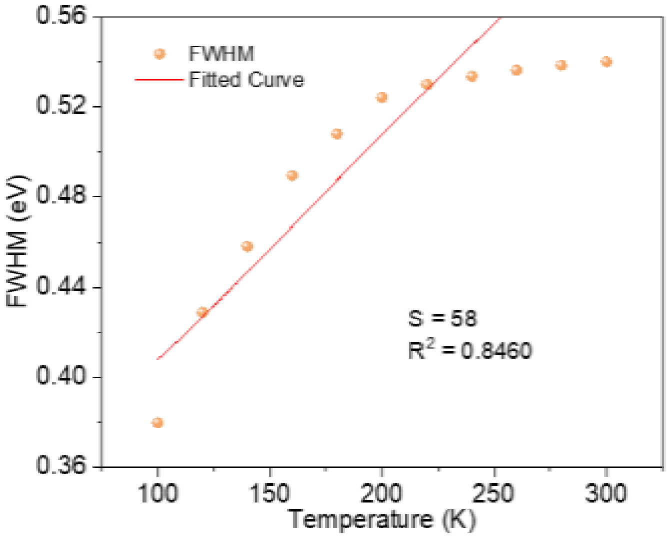

Figure S14. FWHM of PPDCuI PL as a function of temperature.

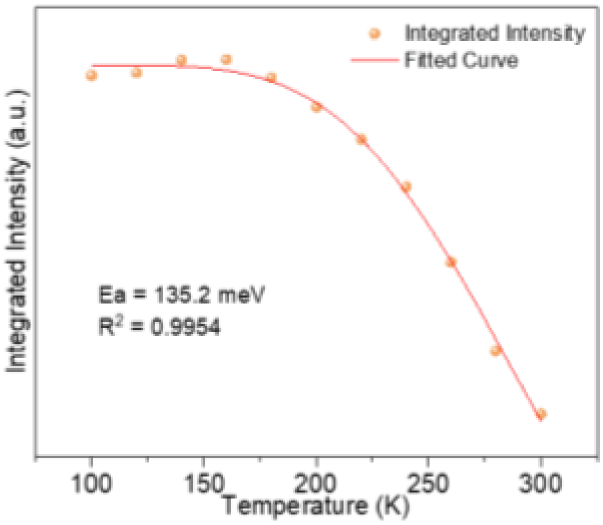

Figure S15. Experimental and fitted integral emission intensity versus temperature of PPDCuI.

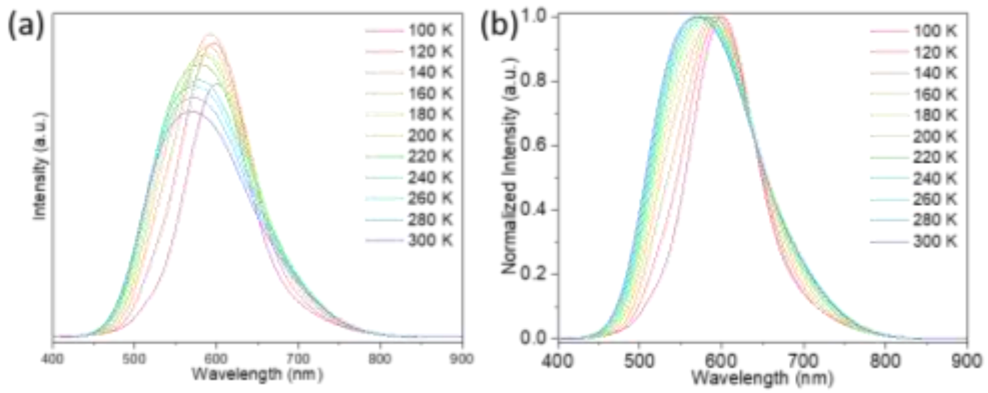

Figure S16. (a) Temperature dependent RL spectra of PPDCuI (T = 100 K – 300 K). (b) Normalized Temperature dependent RL spectra of PPDCuI (T = 100 K – 300 K).

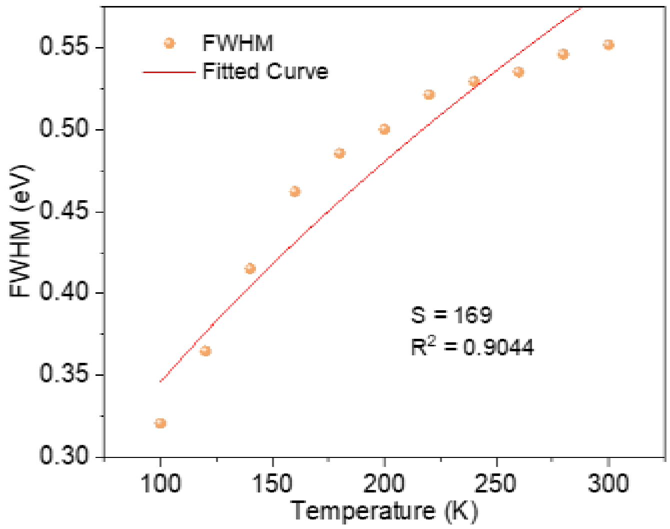

Figure S17. FWHM of PPDCuI RL as a function of temperature.

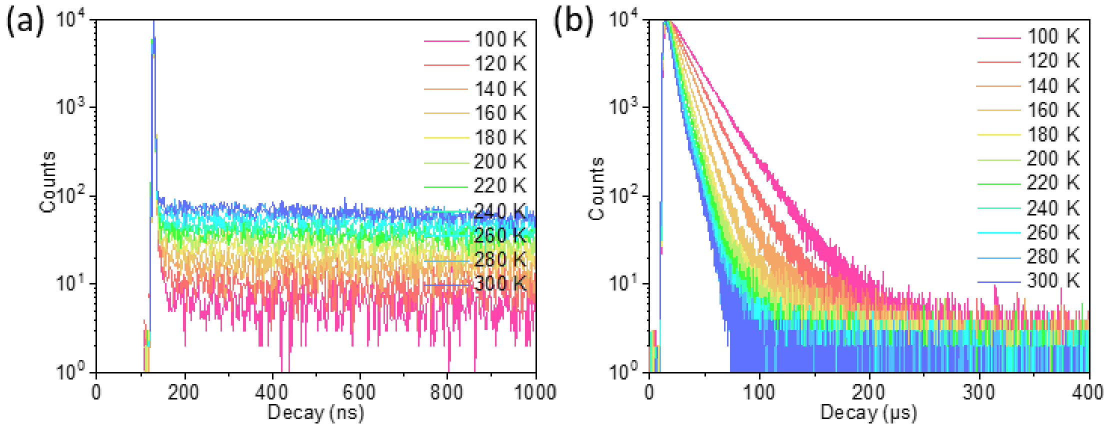

Figure S18. TDPL decay curves of PPDCuI singlet states (a) and triplet states (b) monitored at 550 nm (T = 100 K – 300 K).

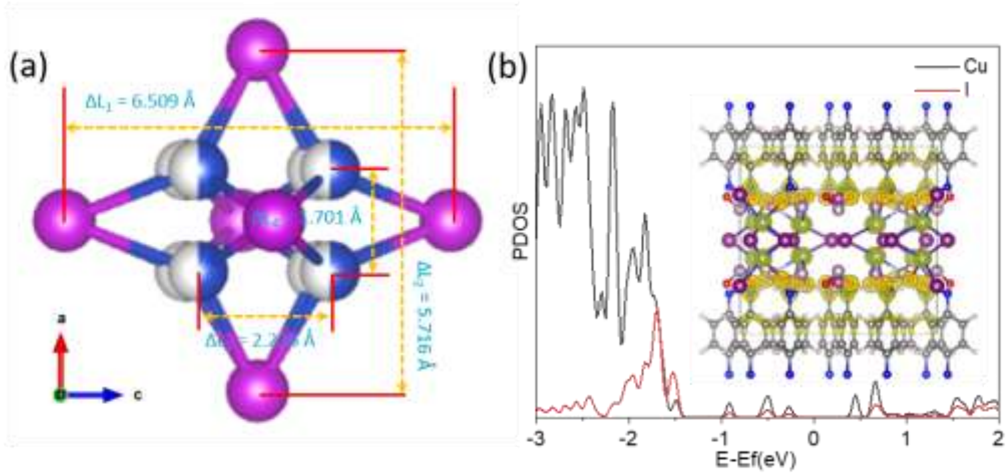

Figure S19. (a) Distances between the farther I-I and Cu-Cu for [Cu_4I_6] core in PPDCuI. (b) Partial density of states (PDOS) of PPDCuI. The insert is the partial charge densities in the energy ranges of [-1， 0].

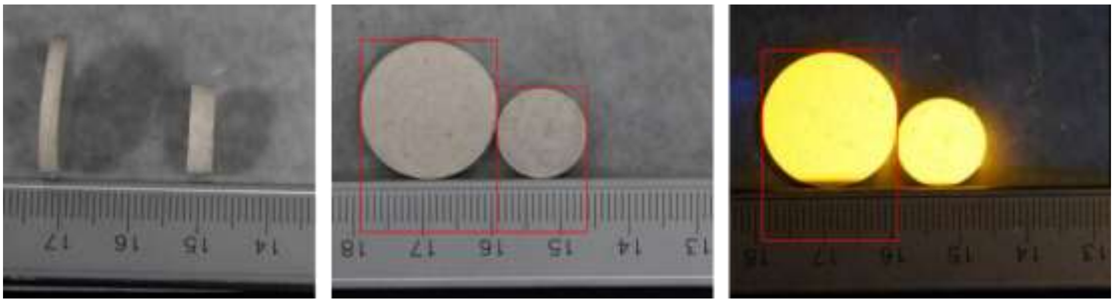

Figure S20. Wafers of PPDCuI with different sizes.

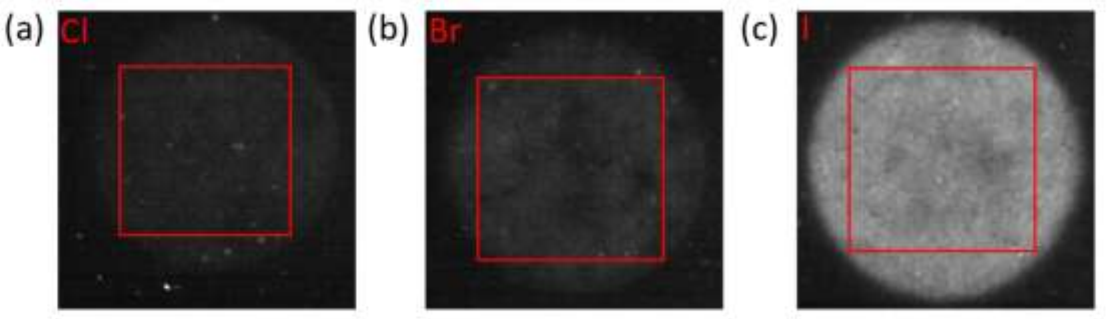

Figure S21. The grayscale images of the wafer samples taken by the camera under fast neutron irradiation. From left to right are $PPDH_2CuCl$ (a), $PPDH_2CuBr$ (b), and PPDCuI (c) in sequence.

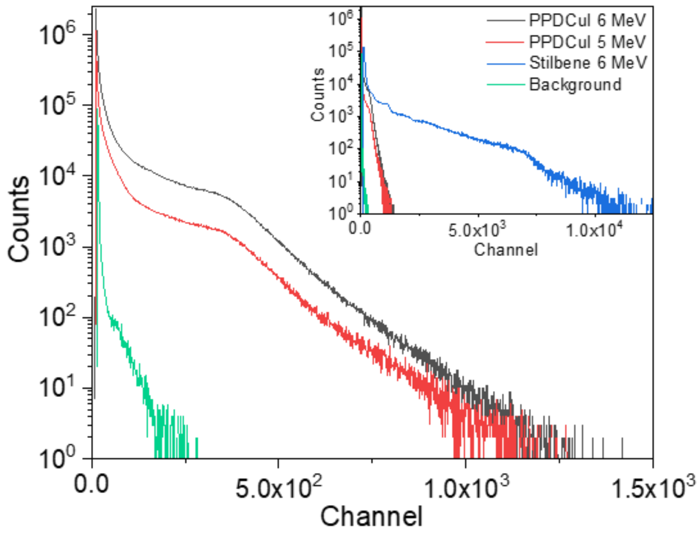

Figure S22. Amplitude spectra of PPDCuI wafer, Stilbene, and background.

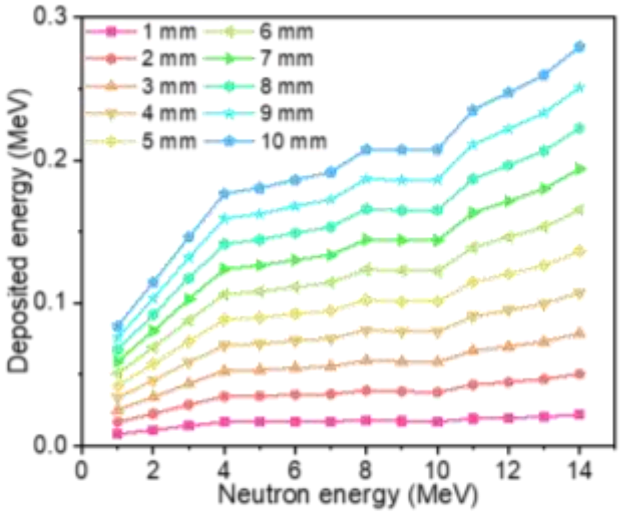

Figure S23. Deposited energies of different thicknesses (1-10 mm) versus neutron energies (1-14 MeV).

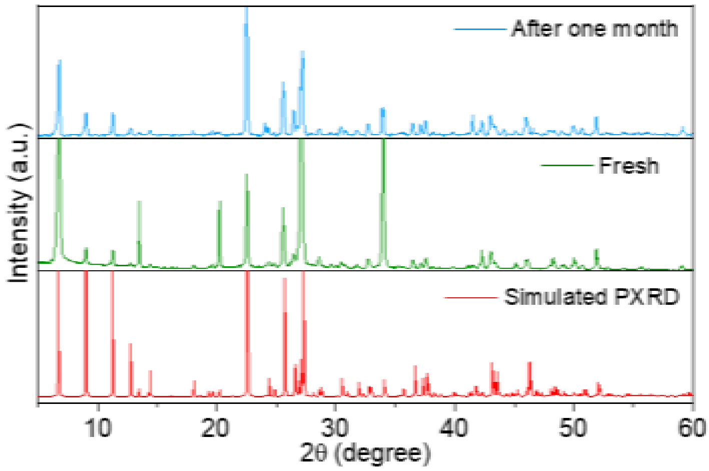

Figure S24. Comparison PXRD of fresh samples and those that have been placed in the air environment for one month using simulated PXRD.

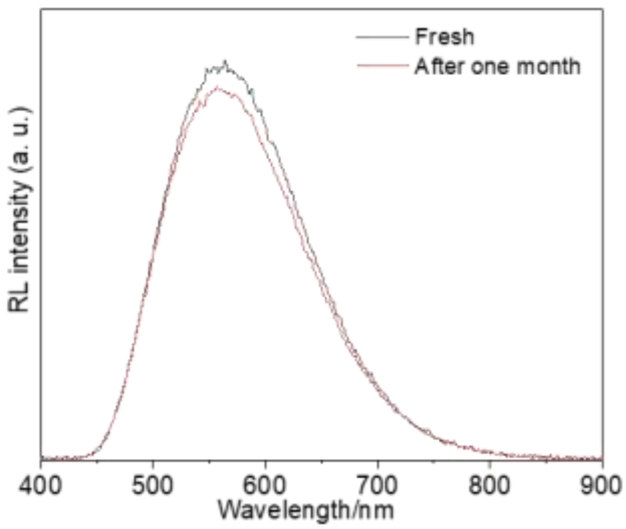

Figure S25. Comparison RL spectra of fresh samples and those that have been placed in the air environment for one month.

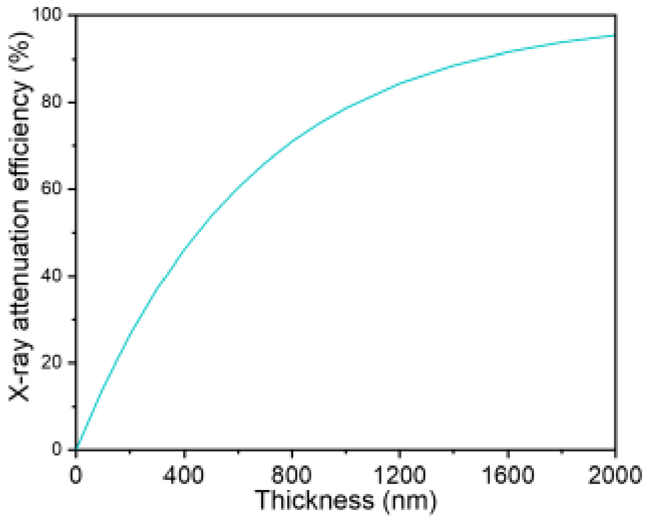

Figure S26. Attenuation efficiencies of PPDCuI scintillator as a function of thickness at 30 keV.

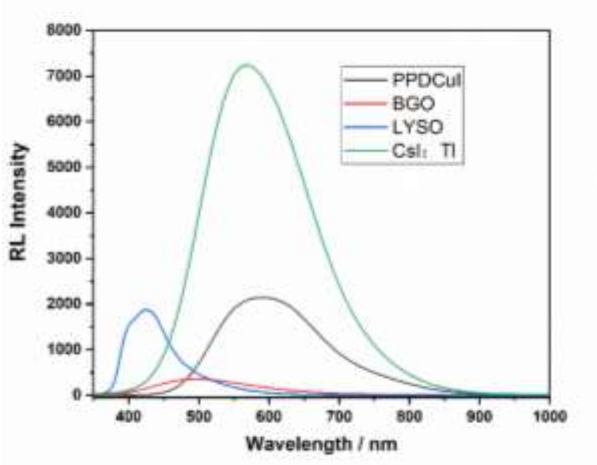

Figure S27. RL spectra of PPDCuI, BGO, LYSO, and CsI:Tl.

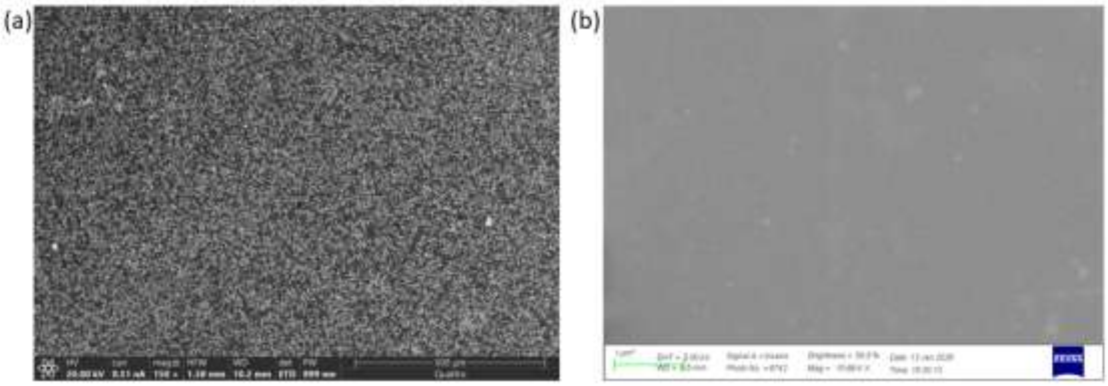

Figure S28. SEM images of PPDCuI@PMMA film at 20 kV (a) and 5 kV (b) measurement voltages.

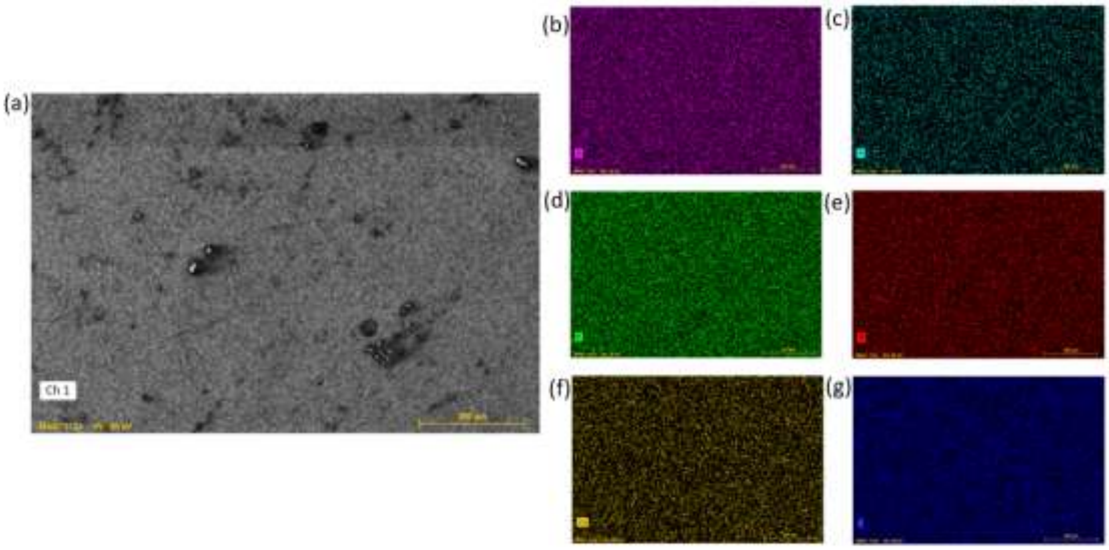

Figure S29. EDS mapping of PPDCuI@PMMA film for the selected area.

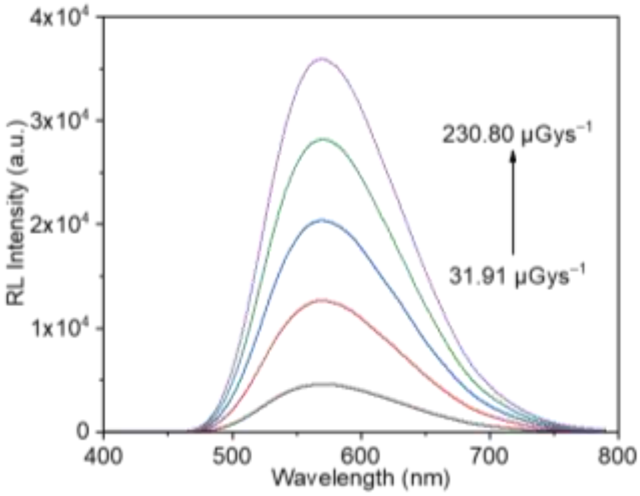

Figure S30. RL spectra of PPDCuI@PMMA scintillator irradiation under different X-ray doses.

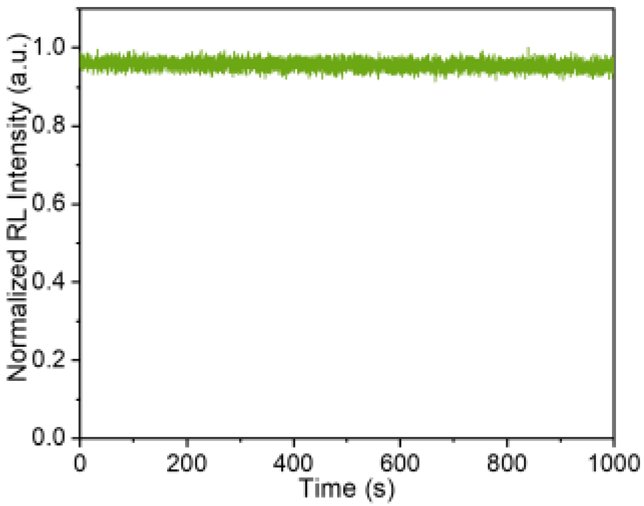

Figure S31. The stability of PPDCuI@PMMA scintillator under 30 on/off cycles upon X-ray irradiation with a dose rate of 647.14 mGy_{air} s^{-1}.

Table S1. The advantages and disadvantages of fast neutron detection vs X-ray detection.

Comparative dimension	Fast neutron detection	X-ray detection
**Principles**	Fast neutrons are typically detected indirectly by measuring the kinetic secondary charged particles generated through elastic or inelastic scattering between fast neutrons and light elements (e.g., hydrogen).	X-rays can directly interact with matter through the photoelectric effect, Compton scattering, or pair production. In detectors, they are directly absorbed and converted into measurable signals.
**Penetrability**	Fast neutrons possess strong penetration and are not easily absorbed by dense materials (such as metals), making them suitable for detecting substances concealed behind heavy shielding.	The penetration of low-energy X-rays is relatively weak, and high-Z materials (such as lead) can effectively shield them. The penetrating ability of X-rays is directly proportional to photon energy: the higher the energy (shorter wavelength), the stronger the penetration. For instance, hard X-rays (0.01–0.1 nm, 10–120 keV) exhibit strong penetration, making them suitable for medical CT imaging and industrial inspection, whereas soft X-rays (0.1–10 nm, 0.1–10 keV) exhibit limited penetration and are primarily used for monitoring lightweight objects or surface-sensitive analysis.
**Spatial resolution**	The strong penetration of fast neutrons necessitates the use of thick scintillators, which inevitably induces self-absorption. Moreover, the energy scattering of fast neutrons broadens the excitation region within the scintillator, leading to blurred imaging edges and a reduced spatial resolution (~1 mm).[8]	With high spatial resolution (10-20 lp/mm), it can be used for high-precision imaging.[9]
**Identification capability**	It enables the distinction between light elements (e.g., H, C, N, O) and high-Z	It exhibits high sensitivity to high-Z materials, while showing a weak response

	elements.	to light elements.
**Background interference**	Although the background level of fast neutrons in the environment is low, the concomitant and secondary X/γ-rays can adversely affect fast neutron imaging.	It is highly susceptible to interference from environmental X-rays and background radiation.
**Detector**	Special detectors enriched in hydrogen, such as organic and plastic scintillators, are required; however, they are relatively expensive and available only in limited supply.	A variety of detectors, including scintillators, semiconductors, and gas detectors, have been developed, offering mature technology and relatively low cost.
**Security**	Fast neutrons can cause significant damage to biological tissues; therefore, effective shielding is essential.	X-rays exhibit ionizing radiation effects; however, they can be effectively shielded using lead protection.
**Applications**	Applications include nuclear security (e.g., detection of explosives and special nuclear materials), oilfield logging, cosmic ray research, and nuclear reactor monitoring.	Medical imaging, industrial non-destructive testing, and security screening.

Table S2. The reported crystal structure parameters (Chem. Sci. 2023, 14, 5415, J. Mater. Chem. C, 2024, 12, 17587) and our result.

	**Chem. Sci. 2023, 14, 5415**			J. Mater. Chem. C, 2024, 12, 17587	**Our Result**
**Chemical formula**	$C_{12}H_{26}N_4 Cu_2Cl_3 O_6P_3$	$C_{12}H_{26} N_4 Cu_2Br_3 O_6P_3$	**$C_{12}H_{20}N_4 Cu_2I_3 O_6P_3$**	$C_{24}H_{40}N_8 Cu_4I_6 O_{12}P_6$	**$C_{12}H_{20}N_4 Cu_2I_3 O_6P_3$**
**Abbreviation**	$DPCu_4Cl_6$	$DPCu_4Br_6$	$DPCu_4I_6$		PPDCuI
**Formula weight**	648.71	782.09	917.01	1834.02	917.01
**Temperature/ K**	173.00	173.00	193.00	293	120.00
**Crystal system**	orthorhombic	tetragonal	tetragonal	orthorhombic	tetragonal
**Space group**	Ccce	I41/acd	P4/m	Pbam	P 4/m
***a*/Å**	13.6165(6)	13.6690(6)	13.8592(5)	13.926 (1)	13.814(2)
***b*/Å**	25.2859(10)	13.6690(6)	13.8592(5)	13.9954 (10)	13.814(2)
***c*/Å**	13.6152(5)	13.6152(5)	13.1579(7)	13.2077 (10)	13.146(4)
**α/°**	90	90	90	90	90
**β/°**	90	90	90	90	90
**γ/°**	90	90	90	90	90
**Volume/Å3**	4687.8(3)	9588.8(11)	2527.3(2)	2574.2 (3)	2508.7(12)
**ρcalc(g/cm3)**	1.838	2.167	2.410	2.366	2.428
**μ/mm-1**	2.398	7.011	5.572	5.470	5.613
**F(000)**	2624.0	6112.0	1720.0	1720.0	1720

CCDC number	2236271	2236273	2253788	2371988	2401669

Table S3. The temperature-dependent lifetimes of singlet and triplet states.

Temperature (K)	Singlet lifetimes (ns)	Triplet lifetimes (μs)
300	481.38	6.64
280	211.52	7.32
260	167.91	7.73
240	124.36	8.09
220	100.18	7.94
200	91.71	8.74
180	84.35	9.71
160	75.93	11.67
140	43.28	14.38
120	15.79	17.58
100	5.43	21.83

Table S4. The concentration of hydrogen atoms and effective atomic number of commonly used organic, inorganic and organic-inorganic hybrid scintillators.

	**Hydrogen concentration ($\times 10^{22}$ cm^{-3})**	**Z_{eff}**
**Anthrance**	4.22	5.8
**Stilbene**	3.89	5.7
**Poly(vinyltoluene) (PVT)**	5.21	5.7
**Polystyrene (PS)**	5.56	5.7
**Polypropylene (PP)**	7.8	5.4
**PEA_2PbBr_4**	4.31	52.0
**$MAPbBr_3$**	2.7	62.0
**PMA_2PbBr_4**	3.62	52.8
**PEA_2PbCl_4**	4.53	53.9
**$LaBr_3$: Ce**	0	45.0
**$PbWO_4$**	0	73.6
**CsI: Tl**	0	54.0
**Cs_2LiYCl_6: Ce**	0	43.5

The effective atomic number is calculated according to the following formula: $Z_{eff} = \sqrt[2.94]{f_1 \times (Z_1)^{2.94} + f_2 \times (Z_2)^{2.94} + f_3 \times (Z_3)^{2.94} + \cdots}$, where f_n is the fraction of the total number of electrons associated with each element, and Z_n is the atomic number of each element.[10]

Table S5. Energy deposition of PPDCuI (20 mm diameter) at different thicknesses (1-10 mm) and different single-energy neutron energies (1-14 MeV, particle amounts are 500 w).

Neutron energy (MeV)	Diameter (mm)	Thickness (mm)	Total energy deposit (GeV)	Single-particle energy deposition (keV)
1	20	1	41.4734	8.29468
		2	83.7773	16.75546
		3	126.347	25.2694
		4	168.933	33.7866
		5	211.645	42.329
		6	253.864	50.7728
		7	296.051	59.2102
		8	337.536	67.5072
		9	378.622	75.7244
		10	419.203	83.8406
2	20	1	55.9741	11.19482
		2	113.466	22.6932
		3	171.418	34.2836
		4	229.307	45.8614
		5	286.919	57.3838
		6	344.997	68.9994
		7	402.744	80.5488
		8	459.989	91.9978
		9	516.473	103.2946
		10	572.699	114.5398
3	20	1	71.0679	14.21358
		2	144.931	28.9862
		3	218.766	43.7532
		4	292.404	58.4808
		5	366.542	73.3084
		6	439.625	87.925
		7	513.422	102.6844
		8	586.637	117.3274
		9	658.974	131.7948
		10	730.275	146.055
4	20	1	84.6815	16.9363
		2	173.671	34.7342
		3	263.162	52.6324
		4	352.118	70.4236
		5	442.072	88.4144

		**6**	530.462	106.0924
		**7**	618.935	123.787
		**8**	707.147	141.4294
		**9**	795.758	159.1516
		**10**	882.469	176.4938
**5**	**20**	**1**	84.5807	16.91614
		**2**	175.437	35.0874
		**3**	266.572	53.3144
		**4**	357.957	71.5914
		**5**	449.708	89.9416
		**6**	540.671	108.1342
		**7**	631.898	126.3796
		**8**	721.8	144.36
		**9**	812.239	162.4478
		**10**	902.647	180.5294
**6**	**20**	**1**	85.4603	17.09206
		**2**	179.483	35.8966
		**3**	273.427	54.6854
		**4**	367.821	73.5642
		**5**	462.823	92.5646
		**6**	556.301	111.2602
		**7**	651.16	130.232
		**8**	745.364	149.0728
		**9**	838.694	167.7388
		**10**	931.435	186.287
**7**	**20**	**1**	85.9592	17.19184
		**2**	181.729	36.3458
		**3**	278.748	55.7496
		**4**	375.962	75.1924
		**5**	473.69	94.738
		**6**	571.662	114.3324
		**7**	668.33	133.666
		**8**	766.428	153.2856
		**9**	862.779	172.5558
		**10**	958.177	191.6354
**8**	**20**	**1**	90.742	18.1484
		**2**	194.237	38.8474
		**3**	299.503	59.9006
		**4**	405.452	81.0904
		**5**	511.183	102.2366

		**6**	618.01	123.602
		**7**	722.314	144.4628
		**8**	829.325	165.865
		**9**	934.229	186.8458
		**10**	1037.65	207.53
**9**	**20**	**1**	87.5465	17.5093
		**2**	191.187	38.2374
		**3**	296.031	59.2062
		**4**	401.731	80.3462
		**5**	507.394	101.4788
		**6**	613.849	122.7698
		**7**	720.329	144.0658
		**8**	824.413	164.8826
		**9**	930.22	186.044
		**10**	1036.17	207.234
**10**	**20**	**1**	84.727	16.9454
		**2**	188.625	37.725
		**3**	293.53	58.706
		**4**	399.412	79.8824
		**5**	505.34	101.068
		**6**	612.504	122.5008
		**7**	719.409	143.8818
		**8**	824.95	164.99
		**9**	931.186	186.2372
		**10**	1038.15	207.63
**11**	**20**	**1**	96.0734	19.21468
		**2**	214.309	42.8618
		**3**	333.093	66.6186
		**4**	453.192	90.6384
		**5**	573.641	114.7282
		**6**	695.162	139.0324
		**7**	815.995	163.199
		**8**	934.778	186.9556
		**9**	1054.89	210.978
		**10**	1175.21	235.042
**12**	**20**	**1**	98.8292	19.76584
		**2**	223.582	44.7164
		**3**	348.665	69.733
		**4**	475	95
		**5**	602.117	120.4234

		**6**	730.296	146.0592
		**7**	857.167	171.4334
		**8**	982.7	196.54
		**9**	1109.34	221.868
		**10**	1236.25	247.25
**13**	**20**	**1**	102.178	20.4356
		**2**	234.253	46.8506
		**3**	365.487	73.0974
		**4**	498.14	99.628
		**5**	632.117	126.4234
		**6**	766.524	153.3048
		**7**	900.235	180.047
		**8**	1032.61	206.522
		**9**	1164.99	232.998
		**10**	1298.74	259.748
**14**	**20**	**1**	109.479	21.8958
		**2**	252.105	50.421
		**3**	394.087	78.8174
		**4**	536.217	107.2434
		**5**	681.18	136.236
		**6**	825.784	165.1568
		**7**	969.416	193.8832
		**8**	1112.36	222.472
		**9**	1253.99	250.798
		**10**	1397.41	279.482

Table S6. Comparison of the imaging performance of reported copper-cluster scintillators.

Copper-cluster	Spatial Resolution (lp/mm)	Form
Bz-Cu_5I_7	>20	Film[11]
Cu_6I_8(bu-ted)$_2$	20<br>17	Film[12]<br>Film[13]
CuI(Br-MBA)	22	Film[14]
$(C_{12}H_{24}O_6)_2Na_2(H_2O)_3Cu_4I_6$	24.8	Film[15]
$(C_6H_{10}N_2)_2Cu_2I_3(PO_2)_3$	11.14	Film[16]
[BAPMA]Cu_2Br_5	15.79	Film[17]
(DIET)$_3Cu_3X_3$	11.71	Film[18]
[AEPipz]·$CuBr_3$· Br·H_2O	17.25	Film[19]
[$Cu_4I_4(PPh_2Et)_4$]	19.5	Glass[20]
Cu_8I_{10}(bttmpe)$_2$	17.0	Film[21]
(MTP)$_2Cu_4I_6$-β	>20	Glass[22]
PPDCuI	25.8	Film (This work)

References:

1. Kresse, G.; Furthmuller, J., Efficiency of ab-Initio Total Energy Calculations for Metals and Semiconductors Using a Plane-Wave Basis Set. *Comp. Mater. Sci.* **1996,** 6 (1), 15-50.

2. Kresse, G.; Furthmuller, J., Efficient Iterative Schemes for ab Initio Total-Energy Calculations Using a Plane-Wave Basis Set. *Phys. Rev. B* **1996,** 54 (16), 11169-11186.

3. J., K. G. a. H., Ab-Initio Molecular-Dynamics for Liquid-Metals. *Phys. Rev. B* **1993,** 47, 558-561.

4. Blochl, P. E., Projector Augmented-Wave Method. *Phys. Rev. B* **1994**, 50 (24), 17953-17979.

5. Perdew, J. P.; Wang, Y., Accurate and Simple Analytic Representation of the Electron-Gas Correlation-Energy. *Phys. Rev. B* **1992,** 45 (23), 13244-13249.

6. Perdew, J. P.; Burke, K.; Ernzerhof, M., Generalized Gradient Approximation Made Simple. *Phys. Rev. Lett.* **1996,** 77 (18), 3865-3868.

7. Grimme, S.; Antony, J.; Ehrlich, S.; Krieg, H., A Consistent and Accurate ab Initio Parametrization of Density Functional Dispersion Correction (DFT-D) for the 94 Elements H-Pu. *J. Chem. Phys.* **2010,** 132 (15).

8. Lehmann, E. H.; Mannes, D.; Strobl, M.; Walfort, B.; Losko, A.; Schillinger, B.; Schulz, M.; Vogel, S. C.; Schaper, D. C.; Gautier, D. C.; Newmark, D., Improvement in The Spatial Resolution for Imaging with Fast Neutrons. *Nucl. Instrum. Meth. A* **2021,** *988*, 164809.

9. (a) He, Z.-L.; Li, W.-G.; Chen, J.-H.; Luo, J.-B.; Wei, J.-H.; Peng, Q.-P.; Kuang, D.-B., Melt-Processing Enabled Flexible Metal Halide-Nylon Luminescent Films with Enhanced Optical

Transmission for Curved X-Ray Imaging. *Adv. Funct. Mater.* **2025,**, 2503523; (b) Zhang, M.; Wang, X.; Yang, B.; Zhu, J.; Niu, G.; Wu, H.; Yin, L.; Du, X.; Niu, M.; Ge, Y.; Xie, Q.; Yan, Y.; Tang, J., Metal Halide Scintillators with Fast and Self-Absorption-Free Defect-Bound Excitonic Radioluminescence for Dynamic X-Ray Imaging. *Adv. Funct. Mater.* **2021,** *31* (9), 2007921; (c) Heo, J. H.; Shin, D. H.; Park, J. K.; Kim, D. H.; Lee, S. J.; Im, S. H., High-Performance Next-Generation Perovskite Nanocrystal Scintillator for Nondestructive X-Ray Imaging. *Adv. Mater.* **2018,** *30* (40), 1801743.

10. Murty, R. C., Effective Atomic Numbers of Heterogeneous Materials. *Nature* **1965,** *207* (4995), 398-399.

11. Liang, M.; Gang, K.; Li, L.; Liu, K.; Yan, D.; Wang, S.; Liu, S.; Liu, X.; Zhao, Q.; Zheng, K., Ligand-Engineered All-In-One Cu(I) Iodide Complex Enables Near-Unity Photoluminescence and Advanced 3D X-ray Image Reconstruction. *Angew. Chem. Int. Ed.* **2025,** e202512471.

12. Wang, Y.; Zhang, T.; Zhao, W.; Xu, W.; Wu, Z.; Suh, Y. D.; Zhang, Y.; Liu, X.; Huang, W., Machine Learning-Guided Discovery of Copper(I)-Iodide Cluster Scintillators for Efficient X-ray Luminescence Imaging. *Angew. Chem. Int. Ed.* **2024,** *64* (1), e202413672.

13. Gu, R.; Han, K.; Jin, J.; Zhang, H.; Xia, Z., Surfactant-Assisted Synthesis of Hybrid Copper(I) Halide Nanocrystals for X-ray Scintillation Imaging. *Chem. Mater.* **2024,** *36* (6), 2963-2970.

14. Gu, C.; Wang, T.; Qin, X.; Liu, X., Halogen Bonding-Enabled Stabilization of Copper(I) Cluster Scintillators in Aqueous Environments. *Adv. Opt. Mater.* **2025,** *13* (22), 2501022.

15. Zhao, W.; Wang, Y.; Li, R.; Liu, X.; Huang, W., Flash Synthesis of High-Performance and Color-Tunable Copper(I)-Based Cluster Scintillators for Efficient Dynamic X-ray Imaging. *npj Flex. Electron.* **2024,** *8* (1).

16. Zhao, Y.; Chen, D.; Tang, H.; Liu, H.; Liu, Y.; Dang, Y.; Lin, Q., Cuprous-Based Layered Single-Crystalline Scintillators for X-ray Detection and Imaging. *J. Mater. Chem.C* **2024,** *12* (43), 17587-17594.

17. Liu, Y.-H.; Wang, N.-N.; Ren, M.-P.; Yan, X.; Wu, Y.-F.; Yue, C.-Y.; Lei, X.-W., Zero-Dimensional Hybrid Cuprous Halide of [BAPMA]Cu_2Br_5 as a Highly Efficient Light Emitter and an X-Ray Scintillator. *ACS Appl. Mater. Interfaces* **2023,** *15* (16), 20219-20227.

18. Han, K.; Jin, J.; Su, B.; Qiao, J.; Xia, Z., Promoting Single Channel Photon Emission in Copper(I) Halide Clusters for X-Ray Detection. *Adv. Opt. Mater.* **2022,** *10* (20), 2200865.

19. Lin, N.; Wang, X.; Zhang, H.-Y.; Sun, K.-Q.; Xiao, L.; Zhang, X.-Y.; Yue, C.-Y.; Han, L.; Chen, Z.-W.; Lei, X.-W., Zero-Dimensional Copper(I) Halide Microcrystals as Highly Efficient Scintillators for Flexible X-ray Imaging. *ACS Appl. Mater. Interfaces* **2024,** *16* (31), 41165-41175.

20. Dong, C.; Song, X.; Hasanov, B. E.; Yuan, Y.; Gutiérrez-Arzaluz, L.; Yuan, P.; Nematulloev, S.; Bayindir, M.; Mohammed, O. F.; Bakr, O. M., Organic–Inorganic Hybrid Glasses of Atomically Precise Nanoclusters. *J. Am. Chem. Soc.* **2024,** *146* (11), 7373-7385.

21. Ni, J.; Cao, Q.; Xiao, K.; Gang, K.; Liu, S.; Liu, X.; Zhao, Q., Colloidal Copper(I) Iodide Cluster-Based Scintillators for High-Resolution X-Ray Imaging. *Laser & Photonics Rev.* **2024,** *19* (2), 2400963.

22. Li, B.; Jin, J.; Liu, X.; Yin, M.; Zhang, X.; Xia, Z.; Xu, Y., Multiphase Transformation in Hybrid Copper(I)-Based Halides Enable Improved X-ray Scintillation and Real-Time Imaging. *ACS Mater. Lett.* **2024,** *6* (4), 1542-1548.
